# Aggregation-Modulated Excited-State Intramolecular Proton Transfer and Antifungal Potential of Selected 1,3,4-Thiadiazole Derivatives

**DOI:** 10.3390/ijms27177631

**Published:** 2026-08-26

**Authors:** Iwona Budziak-Wieczorek, Klaudia Rząd, Mateusz Koselski, Andrzej Górecki, Magdalena Kachel-Górecka, Alicja Matwijczuk, Bożena Gładyszewska, Patrik Burg, Sylwia Okoń, Arkadiusz Matwijczuk

**Affiliations:** 1 Department of Chemistry, Faculty of Food Sciences and Biotechnology, University of Life Sciences in Lublin, Akademicka 15, 20-950 Lublin, Poland; iwona.budziak@up.edu.pl; 2 Department of Biophysics, Faculty of Environmental Biology, University of Life Sciences in Lublin, Akademicka 13, 20-950 Lublin, Poland; klaudia.rzad@up.edu.pl (K.R.); alicja.matwijczuk@up.edu.pl (A.M.); bozena.gladyszewska@up.edu.pl (B.G.); 3 Department of Plant Physiology and Biophysics, Institute of Biological Sciences, Faculty of Biology and Biotechnology, Maria Curie-Skłodowska University, Akademicka 19, 20-033 Lublin, Poland; mateusz.koselski@mail.umcs.pl; 4 Department of Physical Biochemistry, Faculty of Biochemistry, Biophysics and Biotechnology of the Jagiellonian University, Gronostajowa 7, 30-387 Krakow, Poland; andrzej.gorecki@uj.edu.pl; 5 Department of Machine Operation and Production Processes Management, Faculty of Production Engineering, University of Life Sciences in Lublin, Głęboka 28, 20-612 Lublin, Poland; magdalena.kachel@up.lublin.pl; 6 Department of Horticultural Machinery, Faculty of Horticulture, Mendel University in Brno, Valtická 337, 691 44 Lednice, Czech Republic; patrik.burg@mendelu.cz; 7 Institute of Plant Genetics, Breeding and Biotechnology, University of Life Sciences in Lublin, Akademicka 15, 20-950 Lublin, Poland

**Keywords:** 1,3,4-thiadiazole, excited-state intramolecular proton transfer, aggregation-induced emission, dual fluorescence, molecular aggregation, micellar systems, antifungal activity

## Abstract

The spectroscopic and biological properties of two 1,3,4-thiadiazole derivatives, 4-chloro-6-{5-[(3-chlorophenyl)amino]-1,3,4-thiadiazol-2-yl}benzene-1,3-diol (PhTD-ClB) and 4-ethyl-6-[5-(furan-2-yl)-1,3,4-thiadiazol-2-yl]benzene-1,3-diol (FTD-EtB), were investigated using electronic absorption, steady-state fluorescence, resonance light scattering (RLS), and time-correlated single-photon counting (TCSPC) measurements. The experimental analysis was complemented by fluorescence quantum-yield determination, calculation of radiative and non-radiative rate constants, solvatochromic estimation of dipole-moment changes, and evaluation of intermolecular distances using Kasha’s exciton-splitting model. The spectroscopic results support the occurrence of excited-state intramolecular proton transfer (ESIPT) in both derivatives and indicate that this process is facilitated by molecular aggregation. Spectroscopic studies performed in solvents of different polarity, aqueous media, solvent mixtures, and micellar systems were complemented by an assessment of the antifungal activity of both compounds against selected cereal pathogens. Pronounced changes in fluorescence behavior were observed in micellar systems formed by Triton X-100 and sodium deoxycholate. Depending on the medium, the compounds exhibited either a single short-wavelength emission band or dual emission comprising a second, strongly red-shifted band. Dual emission was particularly evident in selected aqueous, low-polarity, and micellar environments. The combined fluorescence and RLS results indicate that molecular aggregation modifies the balance between the enol- and keto-related emission pathways and promotes an AIE-like enhancement of the ESIPT-associated long-wavelength emission. These findings identify PhTD-ClB and FTD-EtB as environmentally responsive ESIPT fluorophores, while the pronounced antifungal activity observed exclusively for FTD-EtB supports its further investigation as a potential antifungal agent.

## 1. Introduction

The growing demand for sustainable food production has increased the need for effective and environmentally safe strategies to control plant diseases. The extensive use of fungicides in crop production has promoted the emergence of resistant plant pathogens and increased concerns regarding environmental contamination and human health. Consequently, the development of new classes of biologically active compounds remains an important challenge in modern agriculture and chemical biology [1,2]. At the same time, the increasing prevalence of resistant bacterial and fungal pathogens highlights the broader need for new classes of biologically active compounds [3,4]. Addressing these challenges requires an interdisciplinary approach combining chemistry, molecular spectroscopy, biology and materials science [5]. It is worth emphasizing that fungal infections draw particular attention, as due to their invasiveness, they constitute a serious problem in clinical treatment, which is currently limited to three basic classes of antifungal agents: azoles, polyenes and echinocandins [6]. Despite considerable advances in medicinal and agricultural chemistry, no fundamentally new class of antifungal agents has been introduced during the past two decades.

In crop production, where the control of cereal pathogens is important, molecules belonging to the 1,3,4-thiadiazole class are still very attractive and are currently the subject of many studies in the production of pesticides and plant protection products [5,7]. Numerous studies have demonstrated the broad biological activity of 1,3,4-thiadiazole derivatives. This broad biological activity has been associated with the presence of the =N–C–S pharmacophore and the aromatic character of the thiadiazole ring, although the biological efficacy, stability, and toxicity of individual derivatives depend strongly on the nature and position of their substituents. Furthermore, the possibility of introducing various ring modifications allows for increasing the effectiveness of these compounds and reducing their toxicity [8]. 1,3,4-Thiadiazole derivatives exhibit pharmacological activity as anti-tubercular [9], anti-inflammatory [10], anticonvulsant [11], anticancer [12], or antioxidant compounds [13]. 1,3,4-Thiadiazole derivatives also exhibit remarkable antimicrobial properties [14].

Owing to their unique photophysical and structural properties, compounds from the 1,3,4-thiadiazole group can be used in various fields of science and industry, including as laser pigments [15], ligands for rare earth ions [16], molecular probes [17] or UV radiation stabilizers [18]. Understanding the photophysical mechanisms responsible for these properties provides valuable insight into the relationship between molecular structure and biological activity.

Both our research and independent literature reports from other research teams around the world confirm that compounds from the 1,3,4-thiadiazole group are characterized by a unique combination of photophysical, crystallographic and biological properties [19,20], which may provide a basis for explaining the unusual activity against plant pathogens of many of their analogs [21]. The characteristic properties of these compounds include, among others, the ability to complex rare earth metal ions [22], tendency to aggregate [23], and the occurrence of a multi-stage phototautomerization mechanism [24], known as excited-state intramolecular proton transfer (ESIPT) [25]. Moreover, very interesting fluorescence phenomena are observed, such as the effect of dual fluorescence induced by a single excitation, the presence of charge transfer states (CT), often accompanying partial twisting of the molecule (so-called TICT) [26,27], formation of excimer systems [28], the aggregation-induced emission (AIE) phenomenon [29], and also emission bypassing the Kasha rule, as shown in, among others, the work of Brancato et al. [30]. It is also worth mentioning one of the important results of long-term research conducted by our team, namely the discovery that some 1,3,4-thiadiazole derivatives show enhanced antifungal activity when combined with polyene antibiotics, particularly amphotericin B (AmB) [31,32]. Such combinations exhibit enhanced antifungal efficacy while reducing the toxicity associated with AmB [33].

**Scheme 1:**
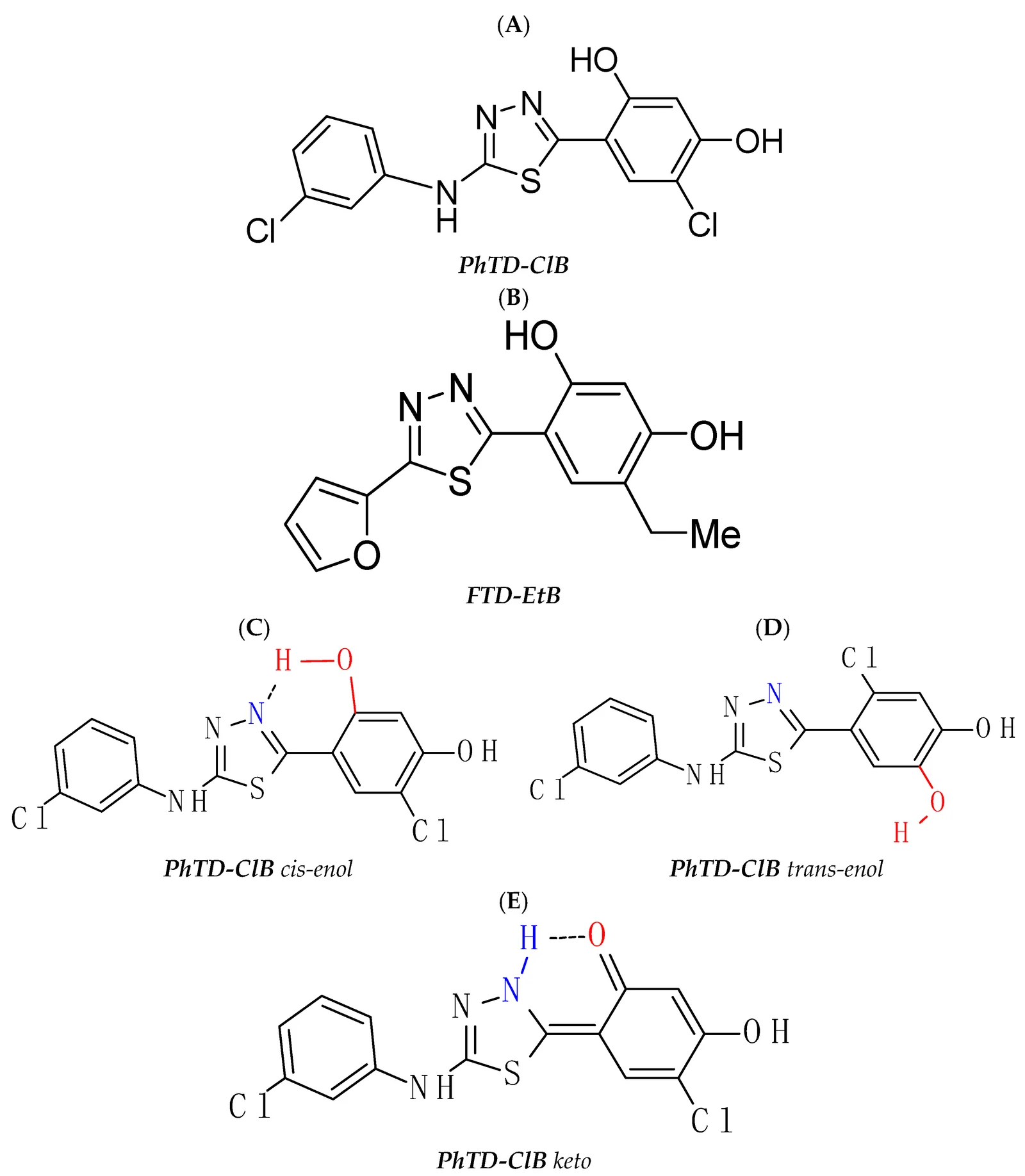
Chemical structures of PhTD-ClB and FTD-EtB and the proposed tautomeric/conformational forms of PhTD-ClB. (**A**) 4-Chloro-6-{5-[(3-chlorophenyl)amino]-1,3,4-thiadiazol-2-yl}benzene-1,3-diol (PhTD-ClB); (**B**) 4-ethyl-6-[5-(furan-2-yl)-1,3,4-thiadiazol-2-yl]benzene-1,3-diol (FTD-EtB); (**C**) cis-enol form of PhTD-ClB; (**D**) trans-enol form of PhTD-ClB; and (**E**) keto form of PhTD-ClB.

**Scheme 2:**
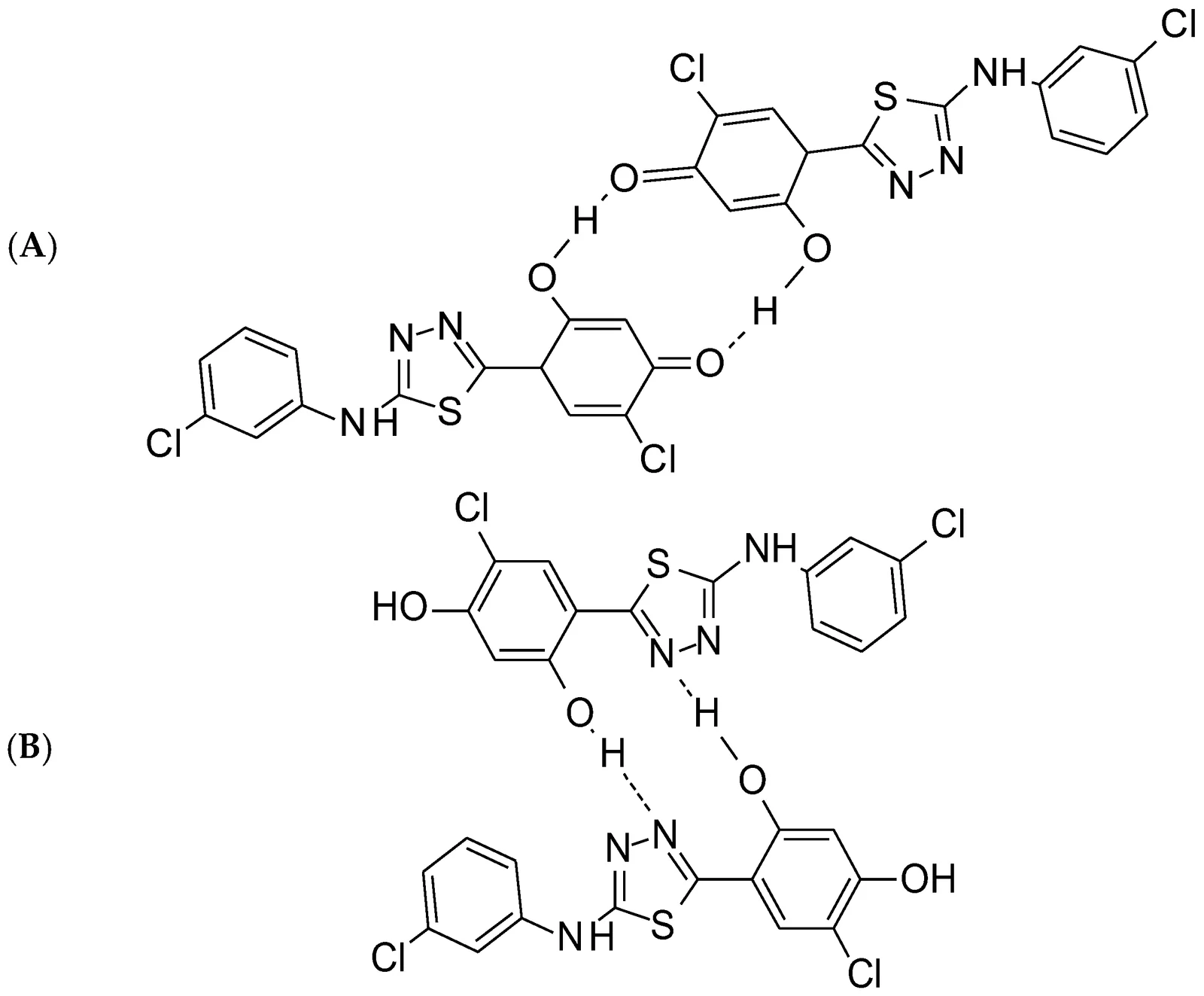
Representative limiting dimeric arrangements of PhTD-ClB: (**A**) head-to-tail and (**B**) card-pack geometries. Both structures are shown as theoretically possible association motifs; the card-pack arrangement was selected for quantitative analysis based on the experimentally observed absorption behavior.

In the present work, comprehensive spectroscopic studies were performed on two structurally related 1,3,4-thiadiazole derivatives: 4-chloro-6-{5-[(3-chlorophenyl)amino]-1,3,4-thiadiazol-2-yl}benzene-1,3-diol (PhTD-ClB) and 4-ethyl-6-[5-(furan-2-yl)-1,3,4-thiadiazol-2-yl]benzene-1,3-diol (FTD-EtB) (Scheme 1 and Scheme 2). Spectral measurements were performed in various solvents of variable polarity, aqueous solutions, and in micellar environments formed from two detergents. The spectroscopic measurements revealed spectroscopic signatures consistent with the enol→keto ESIPT process for both derivatives, manifesting itself, depending on the environment, either as a large spectral shift in the fluorescence emission spectra or the occurrence of dual fluorescence. The ESIPT phenomenon was first described by Weller in 1956 [34], and is currently widely used in many fields, such as laser technologies [35], photovoltaics [36], OLED diodes [37], UV stabilizers in polymers [38], molecular switches [39], protein probes [40] or detection of trace amounts of DNA using FRET measurements [25,41]. One of the related ESIPT mechanisms is also very interesting—the PTTPT mechanism (Proton-Transfer Triggered Proton Transfer), in which one proton transfer initiates another [42].

ESIPT fluorophores frequently exhibit ultrafast proton transfer along a preorganized intramolecular hydrogen bond and large Stokes shifts resulting from substantial structural and electronic reorganization in the excited state. Their fluorescence efficiency, however, depends strongly on the competition between radiative emission and non-radiative relaxation pathways. In selected molecular systems, aggregation or immobilization may restrict intramolecular motions and enhance fluorescence through AIE or AIEE mechanisms, resulting from the restriction of free rotations of molecular fragments—especially around interring bonds—which has been confirmed, among others, in systems embedded in polymer matrices [38]. Importantly, the latest literature reports indicate that the dual fluorescence phenomenon in the ESIPT mechanism occurs only in a rather narrow energy range [43].

Many organic chromophores exhibit reduced fluorescence in the solid state or upon aggregation as a result of aggregation-caused quenching (ACQ) [44]. By contrast, Tang and co-workers reported that selected aromatic compounds exhibiting weak emission in dilute solution became strongly fluorescent upon aggregation [45]. This behavior is known as aggregation-induced emission (AIE) [46]. The AIE mechanism is explained by the concept of restriction of intramolecular motion (RIM) [47], according to which the free rotations of molecular fragments in solution favor non-radiative deexcitation, while their restriction in the condensed phase (e.g., in aggregates) leads to effective light emission [48]. Since the introduction of the AIE concept, numerous molecular systems exhibiting either AIE or aggregation-induced emission enhancement (AIEE) have been reported [49,50]. Analogs of these compounds have potential applications, including in cellular imaging and as biosensors [51], fluorescent sensors [46] and elements of modern optoelectronic and energy devices [52,53]. Accordingly, one of the principal aims of this study was to determine how molecular aggregation affects the fluorescence behavior of the selected thiadiazoles and whether it modulates their ESIPT pathways.

The development of safe and effective strategies for controlling fungal diseases remains a major challenge in modern agriculture [54]. Modern intensive agriculture relies heavily on plant-protection products to limit crop losses caused by fungal pathogens. However, increasing evidence indicates that the extensive use of synthetic xenobiotics in plant-protection products may adversely affect human health and the environment [55]. There is therefore a continuing need to identify new compounds suitable for use as active ingredients in next-generation agrochemicals. It is crucial that these agents are simultaneously used in plant protection strategies against fungal pathogens and do not pose a threat to the environment or humans [56,57]. An alternative or complementary strategy involves compounds capable of activating plant defense mechanisms, including systemic acquired resistance (SAR). Plant defense inducers may therefore support integrated pest-management strategies by reducing reliance on conventional fungicides [58].

PhTD-ClB and FTD-EtB were investigated in solvents of different polarity, aqueous and mixed-solvent media, and micellar systems formed by Triton X-100 or sodium deoxycholate. The experimental approach combined UV–Vis absorption spectroscopy, steady-state fluorescence spectroscopy, resonance light scattering (RLS), and time-correlated single-photon counting (TCSPC). The spectroscopic data were complemented by solvatochromic estimation of ground- and excited-state dipole moments, and calculation of intermolecular distances in dimeric arrangements using Kasha’s exciton-splitting model. Fluorescence quantum yields were determined for both compounds in representative solvent environments. The principal objective was to determine how solvent polarity, hydrogen-bonding ability, and micellar organization influence molecular aggregation, ESIPT-related emission, and dual fluorescence in the two thiadiazole derivatives. In parallel, the antifungal activity of both compounds was evaluated against selected fungal pathogens of cereal crops.

## 2. Results and Discussion

### 2.1. Studies of Spectroscopic Effects of PhTD-ClB and FTD-EtB in Selected Solvents

**Figure 1:**
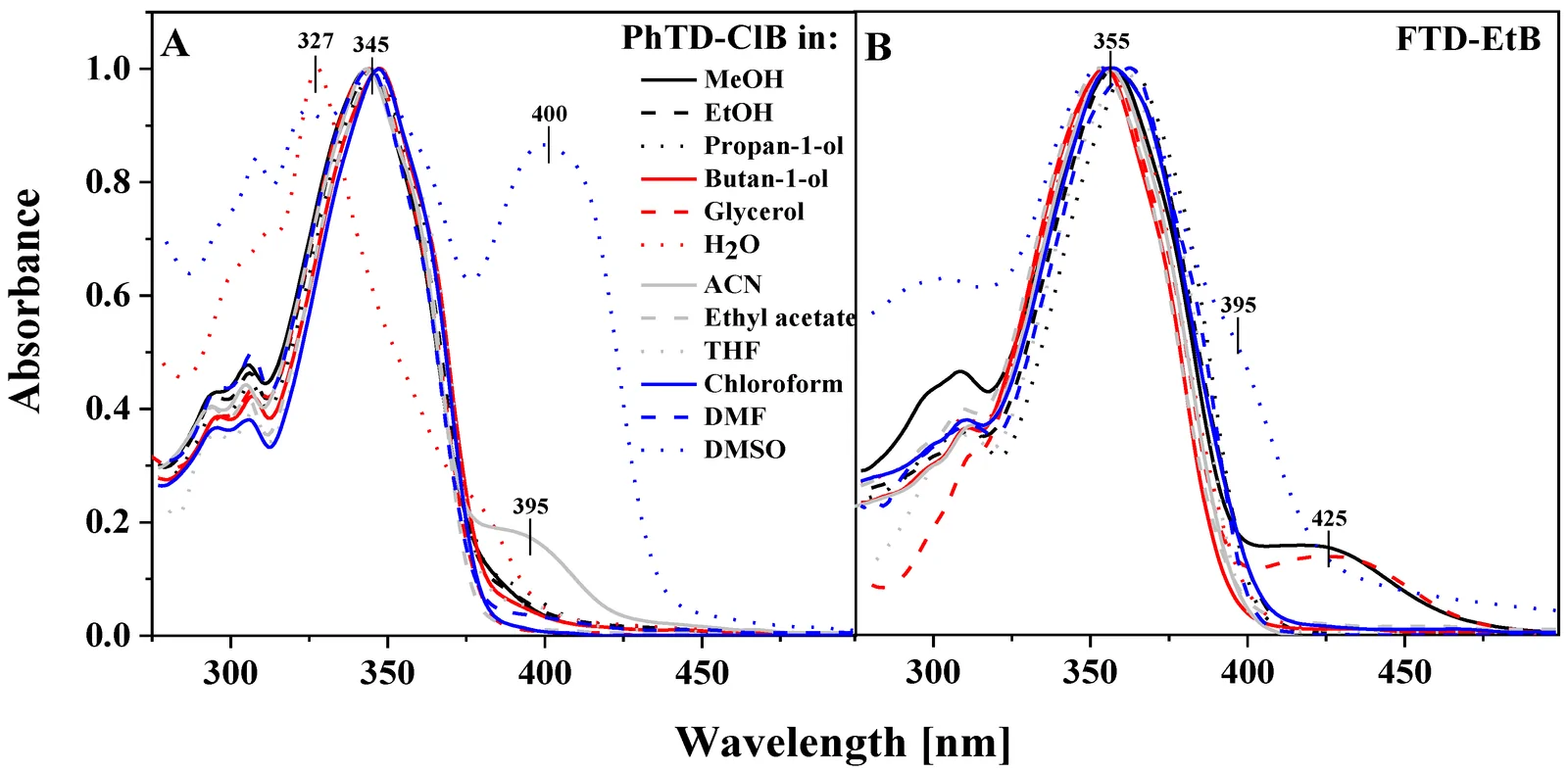
Electronic absorption spectra of PhTD-ClB (Panel (**A**)) and FTD-EtB (Panel (**B**)) recorded in different solvents at constant compound concentrations of 2.95 × 10^−5^ M and 2.40 × 10^−5^ M, respectively.

Figure 1, Panels A and B, present the results of measurements using electron absorption spectroscopy for both compounds selected for the study, i.e., PhTD-ClB (Panel A) and FTD-EtB (Panel B), performed in several selected solvents differing in polarity or ability to form hydrogen bonds. For the remaining solvents used in the study, the values of the absorption maximum position (and fluorescence emission), as well as other parameters used in the work, are presented in Table S1 (in the Supporting Materials (SM)). With the change in solvent polarity, we clearly observe the effect of bathochromic shift for both compounds (Δλ = 20 nm for PhTD-ClB and Δλ = 11 nm for FTD-EtB (Table S1). According to literature reports [59], the observed bathochromic shifts with increasing solvent polarity indicate that the ground states of both compounds are characterized by high polarity [60]. In the case of the presented absorption spectra, we clearly observe quite broad bands related to the S0→S1 transition with a maximum at about 345 nm (Panel A) and 355 nm (Panel B) characteristic of the π→π* electronic transition [42]. At the same time, both compounds exhibit long-wavelength broadening of the main absorption band, with additional spectral features appearing at approximately 365 nm for PhTD-ClB (Panel A) and 385 nm for FTD-EtB (Panel B). The appearance and spectral position of these long-wavelength features are qualitatively consistent with the presence of non-monomeric species, such as dimers or larger aggregates. In the case of PhTD-ClB, an additional long-wavelength feature centered at approximately 425 nm is particularly evident in methanol and glycerol and may indicate the presence of a distinct aggregated species. Because the spectra shown in Figure 1 are normalized and were not recorded at strictly identical molar concentrations, their relative band intensities should not be interpreted as a quantitative measure of the extent of aggregation in different solvents. For PhTD-ClB (Figure 1A), an additional long-wavelength absorption component centered at approximately 425 nm is particularly evident in methanol and glycerol. Rather than being governed solely by bulk solvent polarity, the appearance of this feature is likely associated with specific solute–solvent interactions, particularly hydrogen bonding involving the resorcinol hydroxyl groups and the heteroatoms of the thiadiazole moiety. Such interactions may modify the balance between intramolecular and intermolecular hydrogen bonding and thereby favor a distinct population of associated PhTD-ClB molecules. In glycerol, its high viscosity may additionally restrict molecular reorientation and stabilize such associated species, whereas in methanol, specific and rapidly exchanging hydrogen-bonding interactions may facilitate alternative intermolecular arrangements. Since UV–Vis absorption spectroscopy alone does not provide direct information on aggregate size or geometry, the ~425 nm component is interpreted as evidence consistent with a distinct associated molecular population rather than being assigned unequivocally to a specific higher-order aggregate.

A quantitative determination of solvent-dependent aggregation equilibria would require concentration-dependent absorption measurements under identical conditions and analysis of possible deviations from Beer–Lambert behavior; such measurements were beyond the scope of the present dataset.

The intermolecular arrangement within the aggregates was evaluated using Kasha’s exciton-splitting model:

$$R_{\beta }=\sqrt[{3}]{\frac{\mu^{2}\kappa }{\eta^{2}\beta }}$$

In accordance with the procedure used in our previous studies, μ denotes the transition dipole moment of the interacting molecules, determined from the electronic absorption spectrum in the corresponding solvent by integration of the absorption band; η is the refractive index of the solvent; and β is the dipole–dipole interaction energy. Within the simplified classical approximation adopted here, β was estimated from the difference between the wavenumbers corresponding to the absorption and fluorescence emission maxima in the same solvent, i.e., β = $\left|{\tilde{\nu }}_{abs}-\;{\tilde{\nu }}_{em}\right|$. For the card-pack arrangement, κ = 1, whereas for the head-to-tail arrangement, κ = −2, according to Kasha’s molecular exciton model. According to Kasha’s exciton-splitting theory, the sign and magnitude of the excitonic interaction depend on the relative orientation of the interacting transition dipoles, with card-pack and head-to-tail arrangements representing two limiting geometries. In the present study, the card-pack geometry was selected for quantitative analysis because the experimentally observed hypsochromically shifted absorption components are consistent with excitonic coupling expected for an approximately cofacial arrangement of the chromophores. In contrast, no distinct spectral feature attributable to a head-to-tail arrangement was identified in the absorption spectra under the investigated conditions; therefore, this geometry was not included in the quantitative analysis. Assuming a card-pack arrangement, Kasha’s exciton model yields approximate intermolecular separations of 3.65 Å for PhTD-ClB and 3.77 Å for FTD-EtB. These values are consistent with intermolecular separations previously observed for related thiadiazole derivatives. Nevertheless, because absorption spectroscopy does not provide a direct structural determination of aggregate geometry, these distances should be regarded as model-dependent estimates based on the card-pack arrangement rather than as unequivocal structural parameters of the aggregates present in solution [60,61,62].

The intermolecular separations estimated using the Kasha exciton model, approximately 3.65 Å for PhTD-ClB and 3.77 Å for FTD-EtB, are comparable in magnitude to the close intermolecular contacts observed crystallographically for structurally related resorcinol-substituted 1,3,4-thiadiazole derivatives [19,62]. Such crystal structures demonstrate that closely associated thiadiazole molecules can adopt packing arrangements with intermolecular distances on the order of approximately 3.5–4.0 Å, which is consistent with the 3.65–3.77 Å range obtained from the spectroscopic model. This comparison supports the physical plausibility of the spectroscopically estimated distances and is consistent with molecular arrangements capable of producing appreciable excitonic coupling. Nevertheless, the comparison should be regarded as qualitative, because molecular organization in solution does not necessarily reproduce the packing geometry observed in the crystalline state. Moreover, the simple Kasha point-dipole model provides an approximate description of intermolecular coupling and does not explicitly account for short-range effects, vibronic coupling, or intermolecular charge-transfer contributions that may become important at close intermolecular separations [63]. Therefore, the values of 3.65 and 3.77 Å should be interpreted as approximate spectroscopically derived intermolecular separations rather than direct structural determinations [64].

**Figure 2:**
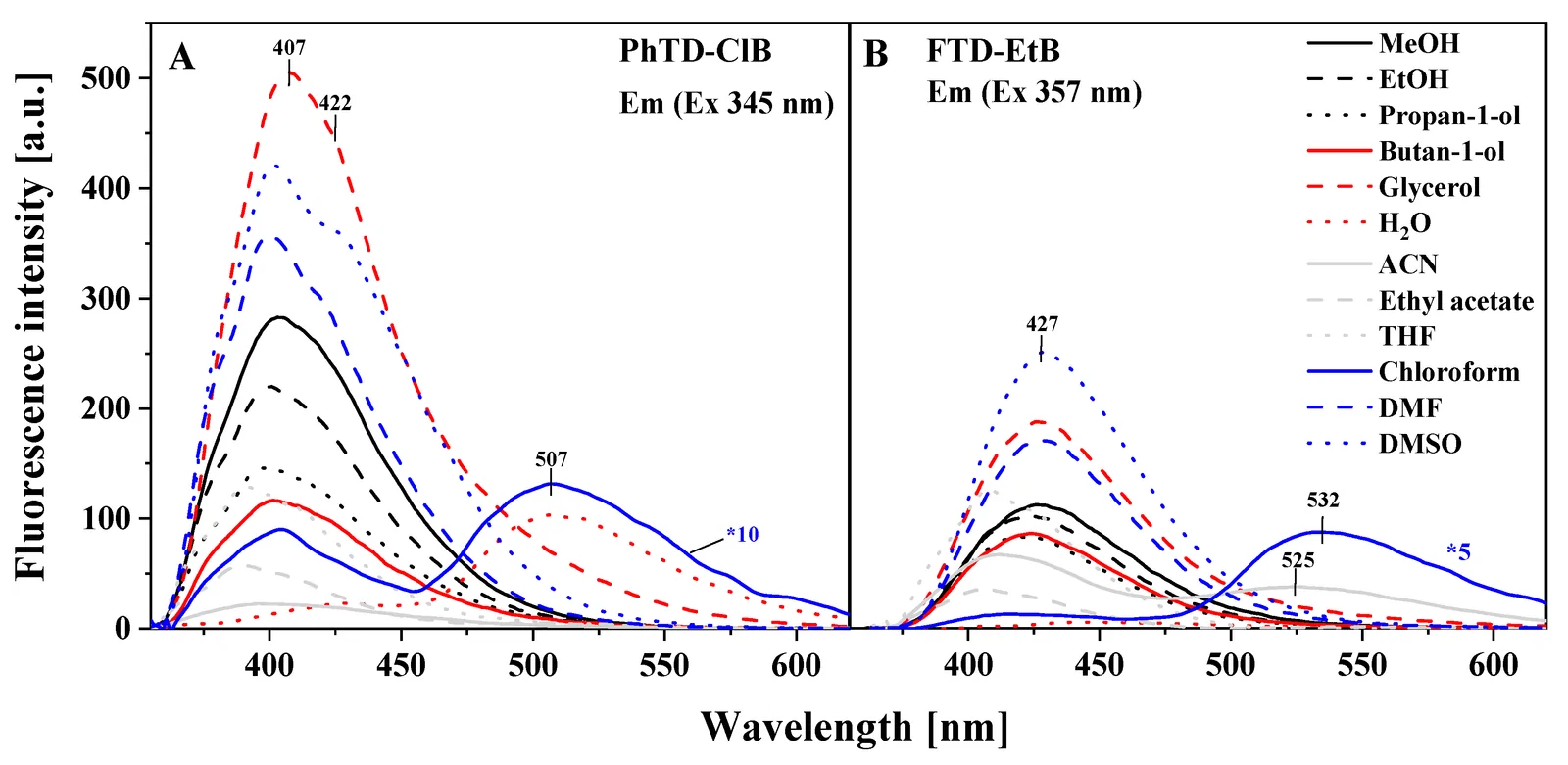
Fluorescence emission spectra of PhTD-ClB (Panel (**A**)) and FTD-EtB (Panel (**B**)) in different solvents at excitation wavelengths corresponding to the main maximum of the absorption spectra in Figure 1. For chloroform, the emission spectrum in panel (**A**) was multiplied by a factor of 10, and in panel (**B**) by a factor of 5.

A particularly interesting phenomenon turned out to be the results presented in Figure 2, panels A and B, illustrating measurements performed using fluorescence spectroscopy for both selected compounds. As can be seen, changing the solvent polarity affects the characteristics of the emission spectra—in most cases, a single fluorescence band was recorded, typical for molecules in an environment of diverse polarity. However, in the case of spectra obtained in water (H_2_O), a clear effect of the so-called dual fluorescence was observed [65]. This makes this result particularly interesting. The change in the polarity of the environment causes a significant shift in the emission band towards longer wavelengths (bathochromic shift), with the emission maximum for the PhTD-ClB compound occurring in the range of ~400–420 nm, while for FTD-EtB it occurs in the range of ~410–430 nm. It is worth emphasizing that all measurements were performed for very low concentrations of the tested compounds (~10^−5^ M). The most distinctive phenomenon in the case of the tested compounds (PhTD-ClB and FTD-EtB) is the occurrence of dual fluorescence in water, although it is accompanied by a clear decrease in the emission intensity. This observation, combined with the characteristic shape of the absorption spectra in H_2_O (Figure 1), suggests the possible involvement of molecular aggregation in the mechanism of dual fluorescence formation. At the same time, in the case of emission measured in chloroform, a dual fluorescence effect also appeared, with one maximum in the range of ~410–430 nm and a second maximum in the range of ~507–532 nm. The significant Stokes shift in the longwave emission suggests a very significant change in the electronic structure of the emitting excited state. When the polarity of the selected medium is reduced, the dual fluorescence effect can be observed for both tested thiadiazoles, PhTD-ClB and FTD-EtB. The dual fluorescence of PhTD-ClB and FTD-EtB in chloroform was considerably weaker than the single-band emission observed in polar solvents, which implies a significantly lower quantum yield of the emitting molecular species in the solvent system. Based on the structural analysis of the studied compounds and reference to the literature, it can be assumed that the observed dual fluorescence effect is related to the presence of the excited-state intramolecular proton transfer (ESIPT) phenomenon. Preliminary interpretation of the results allows us to postulate that the ESIPT mechanism plays a key role in generating dual fluorescence in selected 1,3,4-thiadiazole analogs. Furthermore, the presence of band broadening in the UV-Vis spectra and a significant decrease in emission intensity may also indicate the coexistence of molecular aggregation processes, which additionally influence the photophysical properties of the studied systems. The occurrence of dual fluorescence in both water and chloroform indicates that the ESIPT pathway is not controlled by bulk solvent polarity alone. In chloroform, weak competitive solvation favors preservation of the intramolecular O–H···N hydrogen bond in the cis-enol conformer, thereby providing a direct pathway for excited-state proton transfer. In water, in contrast, strong solvent–solute hydrogen bonding would normally compete with this intramolecular interaction; however, the limited aqueous solubility of the thiadiazole derivatives promotes molecular aggregation. Based on our previous studies of related systems, aggregation modifies the local molecular environment and lowers the energetic barrier for the enol→keto transition, thereby facilitating ESIPT even in the highly polar aqueous phase. Consequently, dual fluorescence observed in water and chloroform can arise from two different microscopic conditions: aggregation-assisted ESIPT in water and preservation of the intramolecular hydrogen bond in chloroform. In solvents of intermediate polarity, stronger solvation combined with a lower tendency toward aggregation favors predominantly the enol*-related emission pathway. Nevertheless, a possible contribution from phenolate species to the long-wavelength emission must also be considered in aqueous and micellar environments. To further distinguish between ESIPT-related keto* emission and emission associated with phenolate species, pH-dependent absorption and fluorescence measurements were performed (Figures S1–S5). The observed spectral changes are consistent with the presence of distinct acid–base forms of the studied thiadiazoles. Under strongly acidic conditions, the spectral behavior is consistent with protonation of a thiadiazole nitrogen and formation of a N-protonated species, whereas at high pH deprotonation of the resorcinolic hydroxyl group is favored, leading to the corresponding phenolate (O^−^) form. In the intermediate and near-neutral pH range, the spectra are consistent predominantly with the neutral hydroxylated form, for which excitation may populate the enol* state followed by ESIPT and formation of the keto* excited state. Importantly, the spectral profiles observed under strongly basic conditions differ markedly from those recorded near neutral pH, indicating that the long-wavelength emission at approximately 505–532 nm under near-neutral conditions cannot be attributed exclusively to phenolate emission. Nevertheless, because aggregation and micellar organization may locally alter the acidity of the resorcinolic OH group, a contribution from phenolate species cannot be completely excluded.

**Figure 3:**
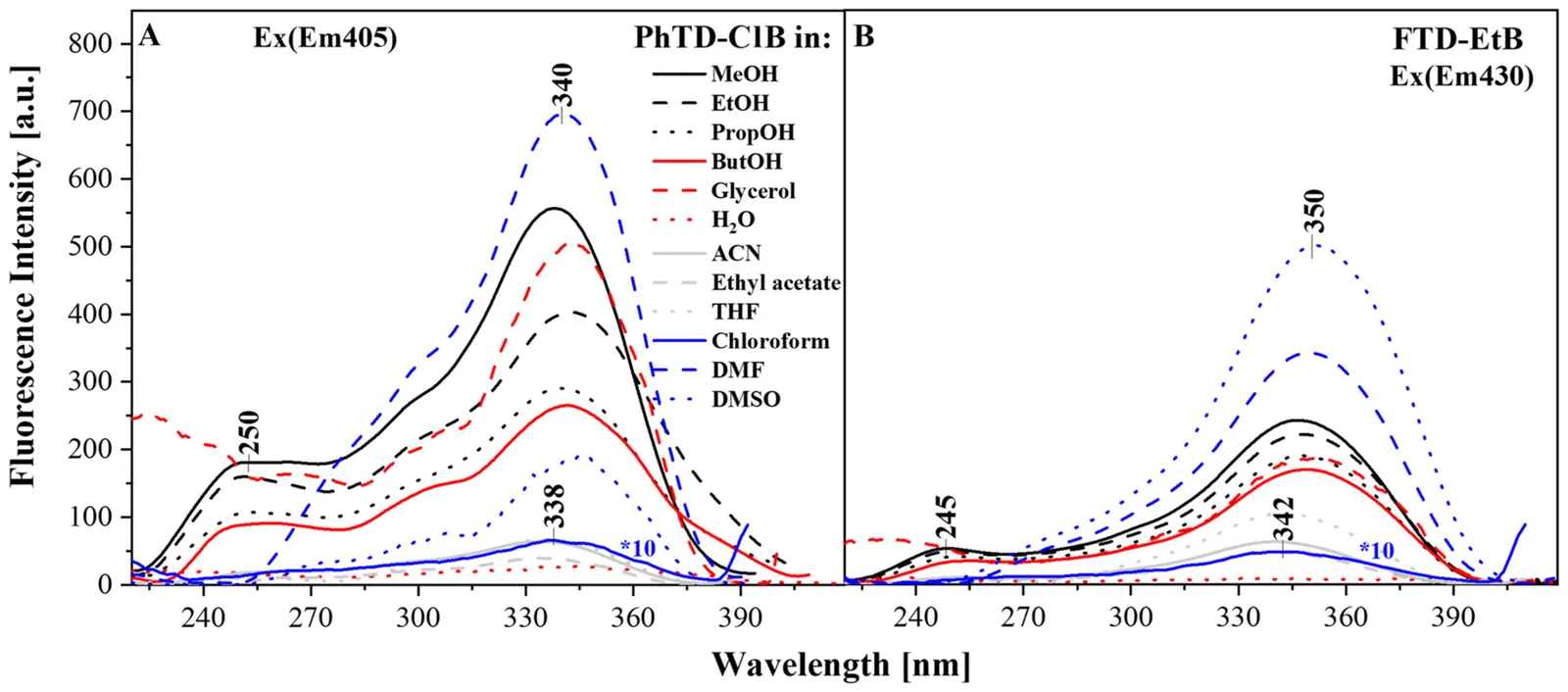
Fluorescence excitation spectra of PhTD-ClB (Panel (**A**)) and FTD-EtB (Panel (**B**)) in different solvents at the emission wavelength corresponding to the maximum of the emission spectra.

In a further study of the ESIPT effect in selected molecules from the 1,3,4-thiadiazole group, fluorescence excitation spectra were measured, as seen in Figure 3. Literature data and our previous studies confirm that the –OH group of the resorcyl ring can be located either closer to the sulfur atom or to the nitrogen atom of the 1,3,4-thiadiazole ring. The existence of appropriate conformations of the molecule enables or blocks the possibility of proton transfer via intramolecular hydrogen bonding. The conformer that possesses an intramolecular hydrogen bond between the hydrogen atom of the –OH group and the nitrogen atom of the thiadiazole ring is called the cis-enol, while the conformer in which the –OH group is located on the sulfur atom side is the trans-enol conformer (Scheme 1). The cis-enol form is more stable than the trans-enol form, which results from the existence of an intramolecular hydrogen bond. Upon photoexcitation, ultrafast intramolecular proton transfer may occur along the O–H···N hydrogen bond in the cis-enol conformer, owing to the increased acidity of the hydroxyl group and basicity of the thiadiazole nitrogen atom [42]. In contrast, the trans-enol form of the molecule, lacking an intramolecular hydrogen bond, emits a single emission that is blue-shifted. In protic solvents, intermolecular hydrogen bonding can disrupt intramolecular hydrogen bonding. In turn, cleavage of the intramolecular hydrogen bond rapidly reduces the population of the cis-enol form, leading to the formation of a solvated enol. This molecule lacks an intramolecular hydrogen bond, so a single fluorescence emission is observed in protic solvents. The measured fluorescence excitation spectra (Figure 3) show a noticeable red shift relative to the corresponding absorption spectra. Analyzing this fact, the excitation spectra for chloroform at 338 nm, for example, can be assigned to the cis-enol form. The observed bathochromic shift in the excitation spectra of the cis-enol compared to the trans-enol is caused by intramolecular hydrogen bonding. It is worth noting that the obtained emission spectra of the keto form are shifted toward the long-wavelength direction relative to the spectra of the enol form, indicating that the energy gap between the first excited state and the ground state must be smaller in the keto* form than in the enol form. Analysis of the absorption spectra in Figure 1 suggests that both compounds exist in the ground state only in the enol form; after emission, the keto* tautomer transforms to the enol form ultrafast [66]. Literature data also confirm our hypotheses, but it is worth emphasizing the important research result presented in Figure 3, which shows broad excitation spectra. Based on M. Kasha’s theory of excitonic splitting and considering the previously mentioned red shift of these spectra, it can be assumed that the observed results are also related to molecular aggregation of the molecules, which in both molecules has a significant impact on the observed results. The hypsochromic shift corresponds to the formation of card-pack aggregates. For excitation spectra obtained in pure methanol or similar solvents, the monomeric form of the compounds may dominate. Taken together, the spectroscopic results support ESIPT as the principal mechanism underlying the observed dual fluorescence; however, in aqueous and micellar environments, a possible contribution from phenolate species cannot be completely excluded. However, as will be discussed below, molecular aggregation can also induce (facilitate) this phenomenon in PhTD-ClB and FTD-EtB molecules.

**Figure 4:**
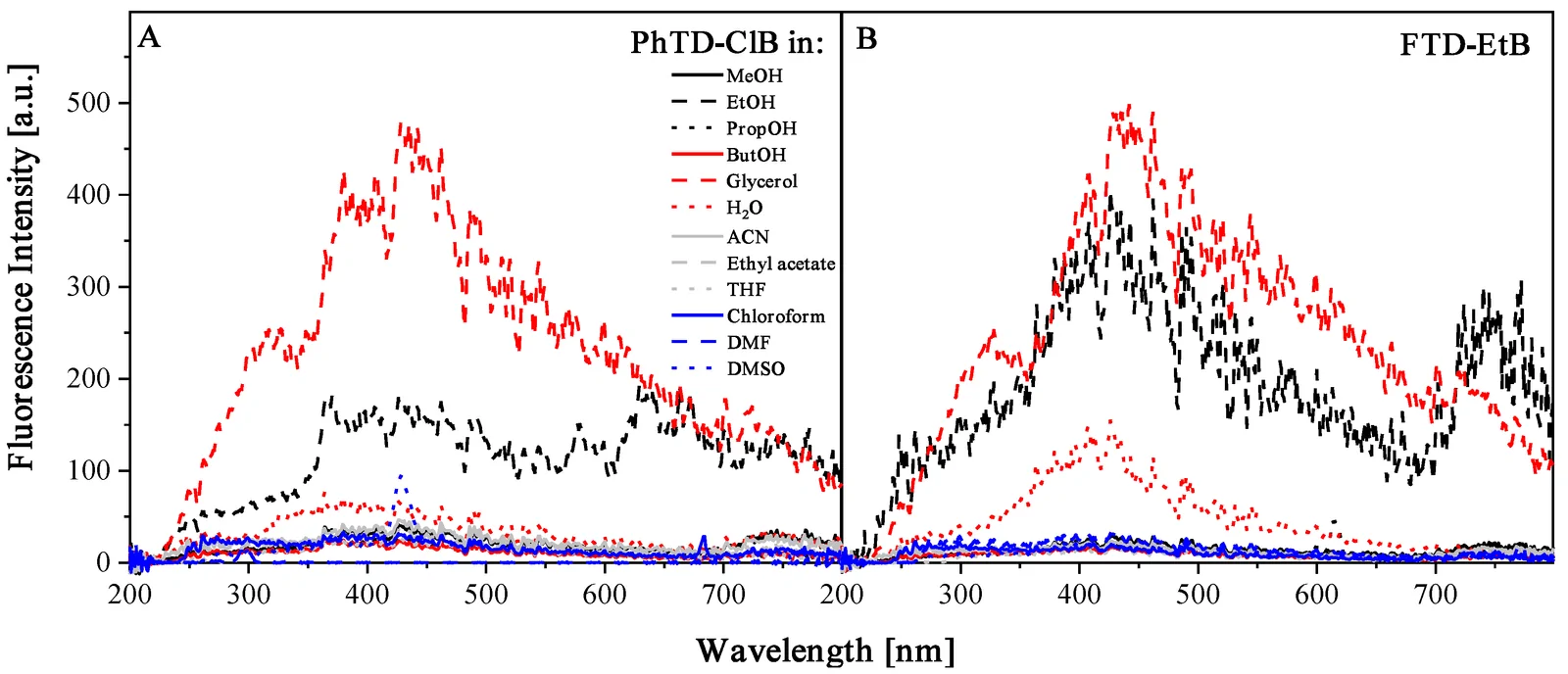
Resonance light scattering spectra of PhTD-ClB (panel (**A**)) and FTD-EtB (panel (**B**)) in different solvents.

In the next step, a clear influence of molecular aggregation effects on the observed fluorescence effects was demonstrated by measuring synchronous RLS (resonance light scattering) spectra. Figure 4 presents the RLS spectra for several solvents of different polarity for both analogs selected for the study, analogously to the above studies in Figure 1, Figure 2 and Figure 3. In accordance with the literature data, here mainly the works of Pasternack and Collings [67], the occurrence of RLS should be associated with the presence of chromophoric aggregation of the molecules present in the solution. However, as can be seen in Figure 4, RLS bands occur both for solvents, where we observe the dual emission effect, and for more polar solvents, where we observe single emission bands, although in their case with noticeably weaker intensity. The effects associated with aggregation will be much more apparent later in this paper. However, it is worth emphasizing at this point that in some solvents (such as water), aggregation effects can play a significant role and facilitate the ESIPT process. The structured RLS profiles may indicate a heterogeneous population of scattering species differing in size or intermolecular organization, although such assignments cannot be made solely on the basis of RLS measurements.

### 2.2. Fluorescence Lifetime Studies in Pure Solvents

Next, the fluorescence lifetimes of PhTD-ClB and FTD-EtB were measured using TCSPC in several different solvents: methanol, propanol, THF, chloroform, ethyl acetate, and DMSO (Figure S6 in the Supplementary Materials). The average fluorescence lifetimes for organic solvents are in the nanosecond range for PhTD-ClB (1.55–2.10 ns), while for FTD-EtB they decrease to the subnanosecond range (0.18–0.60 ns). The polar microenvironment causes a decrease in the average lifetime for both compounds (the lowest value for methanol and ethanol, and the highest for DMSO). The decays could be well-fitted to biexponential models in the case of PhTD-ClB, whereas FTD-EtB required a third component, confirming the coexistence of different emitting states of PhTD-ClB and FTD-EtB and the influence of molecular structure on the spectroscopic properties in the studied solvents (Figure S7 in the Supplementary Materials). Time-resolved fluorescence measurements provide complementary information on the heterogeneity of the excited-state relaxation processes; however, fluorescence decay kinetics alone do not permit an unambiguous assignment of individual lifetime components to specific tautomeric forms. Based on the steady-state emission behavior and our previous studies of structurally related 1,3,4-thiadiazole derivatives, the shorter- and longer-lived components can be tentatively associated with keto- and enol-related excited-state populations, respectively [42].

As shown in Figure S7A, two fluorescence lifetime components are observed for PhTD-ClB in all tested solvents: a shorter one, approximately 0.5–0.7 ns, and a longer one, in the range of approximately 2.0–2.8 ns. At the same time, the fractional contribution of the longer component is larger and amounts to approximately 60–75% of the total emission (Figure S7A). These results indicate that the dominant emission channel for PhTD-ClB is the longer-lived state. Based on previous observations for related systems and the steady-state fluorescence results presented above, the longer-lived component can be tentatively associated with the enol-related emitting population; however, this assignment cannot be considered definitive on the basis of the present TCSPC data alone [68]. In the case of the FTD-EtB compound, fluorescence decays required the use of a three-exponential model (Figure S7B), which indicates a more complex nature of the processes occurring in the excited state. Three lifetime components were observed: very short (approximately 0.1–0.2 ns), intermediate (approximately 0.3–0.6 ns), and longer, exceeding 1 ns. The shortest lifetime component (Figure S2D), which accounts for approximately 60–80% of the total decay amplitude, indicates the dominance of a very fast excited-state relaxation pathway and may tentatively be associated with the keto-related population and/or an intramolecular charge-transfer (ICT) state. The intermediate component (~0.5–1.0 ns) may reflect contributions from the locally excited enol state and/or solvent-relaxation dynamics [69]; however, these assignments remain tentative because the individual emitting species cannot be spectrally resolved from the present TCSPC measurements alone [69]. A definitive assignment of the individual lifetime components would require wavelength-resolved time-resolved measurements, such as TRES or global analysis of fluorescence decays recorded across the emission spectrum, which would enable direct correlation of the decay-associated components with the enol- and keto-related emission bands.

This interpretation is supported by the calculated values of the rate constants for radiative and nonradiative processes. For FTD-EtB, the nonradiative constant *k_nr_* values reach the order of magnitude of 10^9^ s^−1^, while for PhTD-ClB they are approximately 10^8^ s^−1^. This indicates that nonradiative processes occur much more efficiently in FTD-EtB than in PhTD-ClB, leading to shorter mean fluorescence lifetimes and lower emission yields. The observed differences between the compounds tested indicate that modification of the molecular structure significantly affects the course of excited-state deactivation processes. The longer fluorescence lifetimes, the larger contribution of the long-lived emission component, and the lower *k_nr_* values for PhTD-ClB suggest more effective stabilization of the emitting state. In turn, for FTD-EtB the dominance of short-time components and high *k_nr_* values indicate the predominance of fast channels of non-radiative excitation deactivation.

### 2.3. Analysis of Stokes Shifts, Fluorescence Quantum Yields, Radiative and Non-Radiative Decay Constants

**Figure 5:**
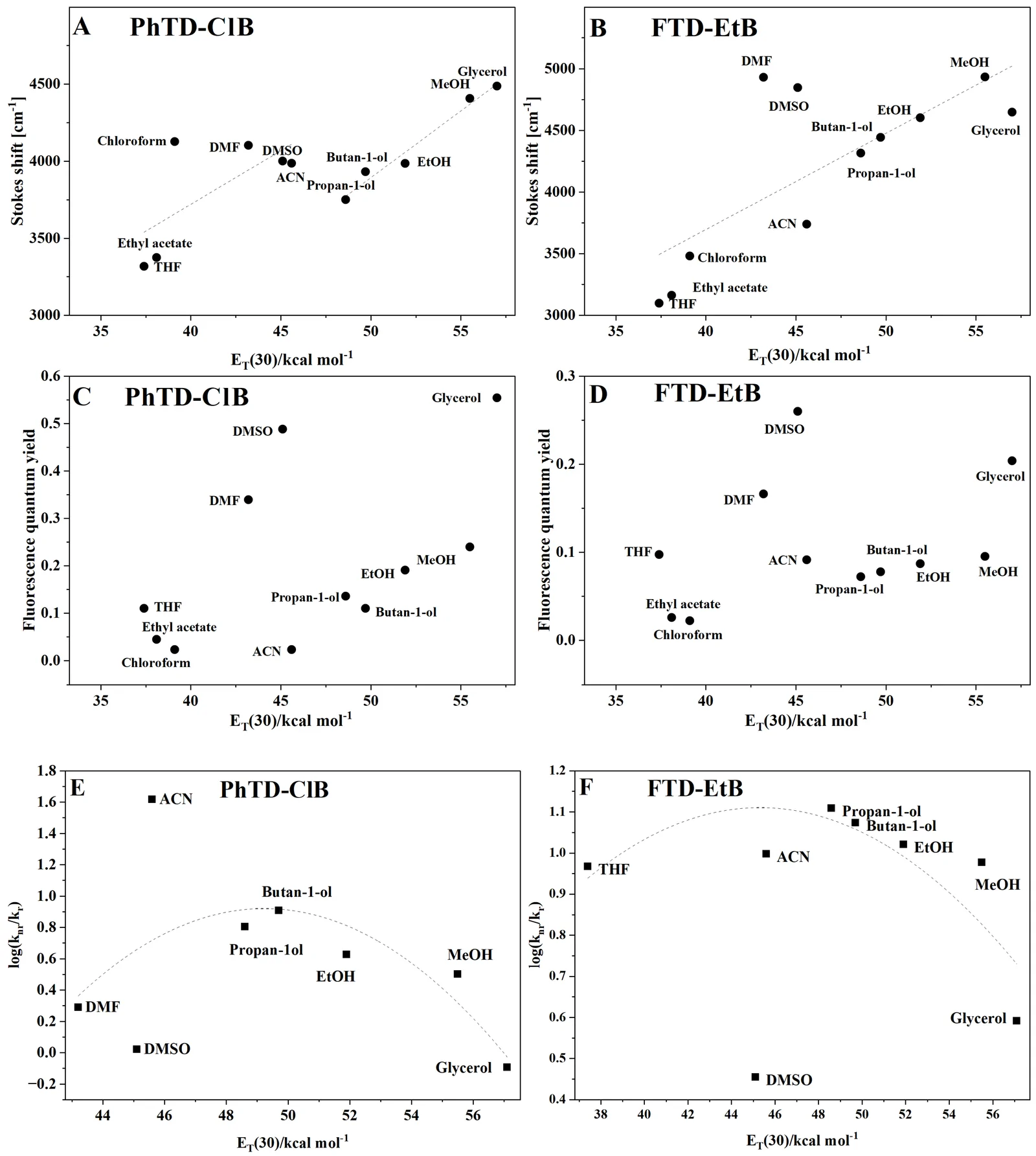
PhTD-ClB Stokes shift (panel (**A**)), fluorescence quantum yields (panel (**C**)) and log(*k_nr_*/*k_r_*) (panel (**E**)) as a function of the solvent’s polarity. FTD-EtB Stokes shift (panel (**B**)), fluorescence quantum yields (panel (**D**)) and log(*k_nr_*/*k_r_*) (panel (**F**)) as a function of the solvent’s polarity.

To complement the experimental studies presented so far, the spectroscopic results were analyzed by calculating the corresponding fluorescence quantum yield values as well as the radiative decay constants *k_r_* and nonradiative *k_nr_* for PhTD-ClB and FTD-EtB in the selected pure solvents. Table 1 and Table 2, as well as Figure 5, show the results of these calculations. It can be observed that the quantum yield *Φ*_F_ is much lower for solvents in which the fluorescence emission spectra showed the presence of dual fluorescence (chloroform), where the keto form dominates (approx. 0.02), compared to solvents for which a single emission associated with enol fluorescence was observed (up to approx. 0.5 for PhTD-ClB and 0.3 for FTD-EtB). The results indicate a significant increase in the *Φ*_F_ value in cases where a single emission band of enol origin dominated. These results indicate that conditions favoring predominantly enol-related emission are associated with higher overall fluorescence quantum yields than conditions in which the long-wavelength keto-related band becomes prominent. It is generally accepted that in some organic systems, such as coumarins (derivatives of 1,2-benzopyrone) or 1,3,4-thiadiazoles, the fluorescence quantum yield tends to be higher in polar solvents [70], which is precisely the reason why the Stokes shift values are best analyzed with respect to the solvent polarity function ET(30) (see Table S2 in the Supplementary Materials). As shown in Figure 5A,B, the Stokes shifts increase with solvent polarity for PhTD-ClB and FTD-EtB. In the case of PhTD-ClB, two linear fits were generated, separately for polar protic solvents (r = 0.98) and for polar aprotic and nonpolar solvents (r = 0.69). In contrast, for FTD-EtB, one linear fit (r = 0.75) was generated for all tested solvents. However, apart from the first fit, a moderate linearity was found for the overall regression, and the actual deviations of the Stokes shift in the tested solvents may result from the influence of hydrogen bonding between the fluorophore molecules and the solvent. The corresponding relationships between *Φ*_F_ and the solvent polarity function ET(30) are presented in Figure 5C,D. The results presented in the figures illustrate that the quantum yields calculated for PhTD-ClB and FTD-EtB molecules in solvents favoring keto* emission (including chloroform) were very low, which fits the specific features of the ESIPT phenomenon.

Next, analysis of the dependence of log(*k_nr_*/*k_r_*) on the solvent polarity function ET(30), presented in Figure 5E,F, indicates that increasing solvent polarity leads to an increase in the relative contribution of radiative processes to the excited state deactivation. This effect is particularly visible for PhTD-ClB, for which the lowest log(*k_nr_*/*k_r_*) values are observed in DMSO and glycerol, corresponding to the highest fluorescence quantum yields. In the case of FTD-EtB, nonradiative processes dominate over the entire polarity range studied, but also, for this compound, the polar environment leads to a partial reduction in their effectiveness.

### 2.4. Estimation of Dipole Moments

On the basis of solvatochromic analyses performed for the tested PhTD-ClB and FTD-EtB systems, the dipole moments in the ground state (μg) and excited state (μe), as well as changes in the dipole moment Δμ, were determined. Calculations were made using the classic Bakhshiev, Kawski–Chamma–Viallet’s models and the microscopic method based on the Reichardt parameter. The fundamental and excited dipole moment were estimated using two methods using two solvent polarity functions F1(ε, n) and F2(ε, n) (Equations (5) and (6)) and the microscopic solvent function (Equation (15)) for various solvents: DMSO, DMF, methanol, ethanol, propan-1-ol, butanol, glycerol, ACN, chloroform, ethyl acetate, THF and water. The values of the refractive index n, dielectric constant ε, and functions F1(ε, n), and F2(ε, n) are presented in Table S2. The calculation results are presented in Figures S8 and S9 and in Table 3.

In Method 1, based on the work of Bakhshiev and Kawski–Chamma–Viallet, for PhTD-ClB and FTD-EtB molecules, the estimated Δµ values for mixed solvents (polar protic, polar aprotic, and nonpolar) are 3.479 and 2.651 D, respectively. For both molecules, moderate-to-good linear correlations were obtained for the two solvent-polarity functions (r^2^ = 0.70–0.88). The remaining deviations from linearity most likely reflect contributions from specific solute–solvent interactions that are not fully captured by the continuum solvent models. For both compounds, the excited-state dipole moment is larger than the ground-state dipole moment, indicating increased molecular polarity upon electronic excitation. The higher Δμ for PhTD-ClB compared to FTD-EtB indicates a stronger charge redistribution in this molecule upon excitation—most likely related to the presence of a more extensive aromatic system and stronger electron-donor/acceptor effects (chloro-phenylamino vs. furanyl). Although PhTD-ClB also exhibits pronounced aggregation-related spectral changes, the present data do not establish a direct causal relationship between the magnitude of Δμ and its aggregation tendency.

Using the Dimroth–Reichardt approach, the changes in dipole moment (Δμ) were estimated to be 2.812 D for PhTD-ClB (r^2^ = 0.58) and 2.637 D for FTD-EtB (r^2^ = 0.70). For FTD-EtB, the Δμ value obtained using this approach is almost identical to that derived from the Bakhshiev/Kawski–Chamma–Viallet analysis (2.637 vs. 2.651 D), indicating good agreement between the two solvatochromic models. In contrast, a larger difference is observed for PhTD-ClB (2.812 vs. 3.479 D). This discrepancy can be attributed to the different descriptions of solvent effects inherent to the two approaches: the Bakhshiev/Kawski–Chamma–Viallet models are primarily based on bulk dielectric and refractive-index properties, whereas the empirical Dimroth–Reichardt parameter is additionally sensitive to microscopic solvent polarity and specific solute–solvent interactions, including hydrogen bonding. Such interactions are particularly relevant for the ESIPT-active PhTD-ClB molecule and can perturb the simple linear relationship between spectral shifts and solvent polarity. Consistently, the Dimroth–Reichardt correlation for PhTD-ClB is relatively weak (r^2^ = 0.58) compared with the Bakhshiev and Kawski–Chamma–Viallet correlations (r^2^ = 0.74 and 0.71, respectively). Therefore, within the present dataset, the Bakhshiev/Kawski–Chamma–Viallet value of Δμ = 3.479 D is considered the more reliable quantitative estimate for PhTD-ClB, whereas the Reichardt-derived value should be regarded as a complementary estimate reflecting the substantial contribution of specific solute–solvent interactions to its solvatochromic behavior.

### 2.5. Studies of Spectroscopic Effects of PhTD-ClB and FTD-EtB in Micellar Systems

**Figure 6:**
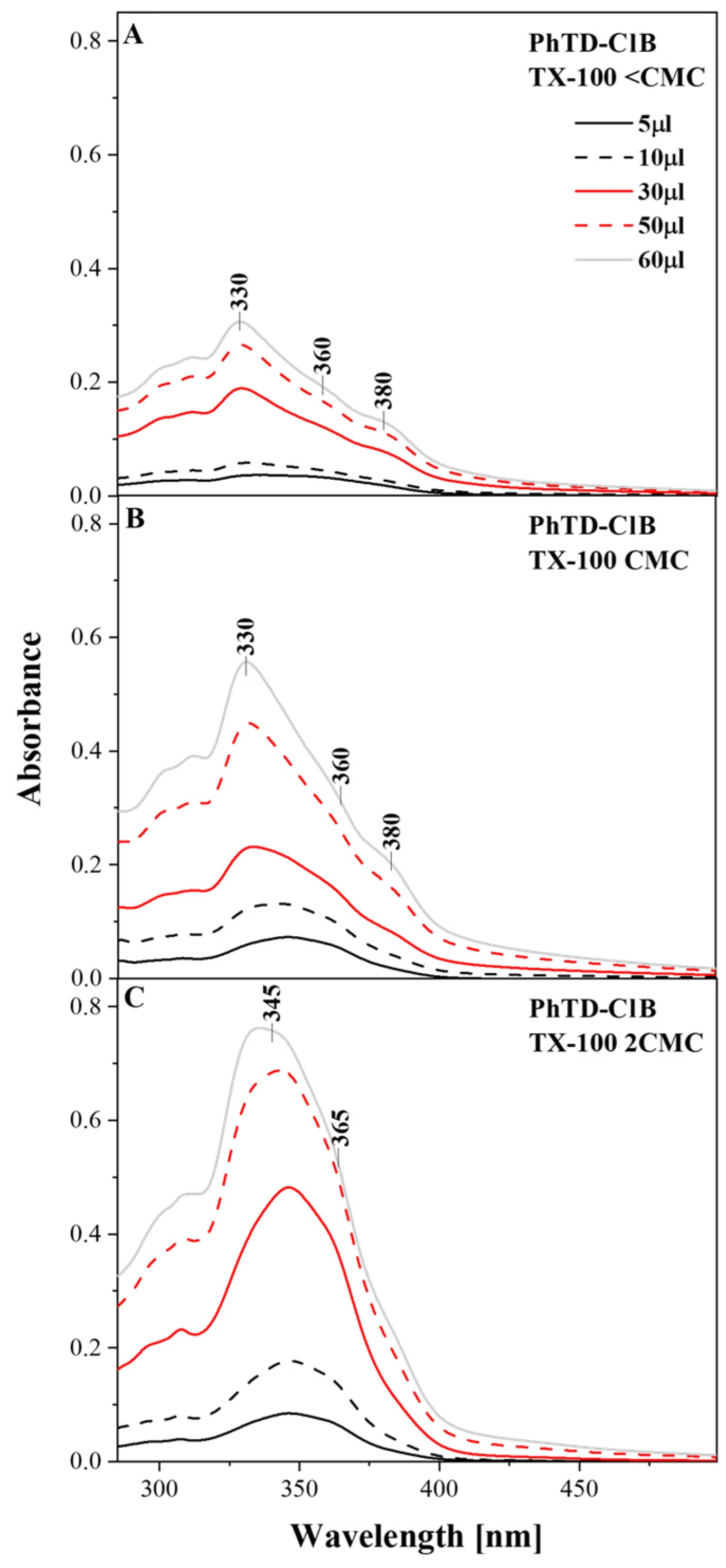
Electronic absorption spectra of PhTD-ClB in the micellar system with concentrations <CMC (panel (**A**)), CMC (panel (**B**)) and 2 CMC (panel (**C**)). The concentrations of the compound are: 4.97 × 10^−6^ M, 9.9 × 10^−6^ M, 2.95 × 10^−5^ M, 4.89 × 10^−5^ M, 5.84 × 10^−5^ M.

To determine how molecular aggregation affects ESIPT and dual fluorescence, both compounds were further investigated in micellar systems formed by TX-100 and NaDC. For this purpose, we used systems that favor this type of phenomenon by changing their hydrophobicity: micellar systems formed with the nonionic surfactant TX-100 and the clinically used ionic sodium salt of deoxycholic acid—sodium deoxycholate (NaDC). Figure 6 shows the UV-visible absorption spectra of the PhTD-ClB compound titrated in the TX-100 micellar system in PBS solution (pH = 7.4). The studies included three surfactant concentrations, corresponding to <CMC, CMC, and 2 CMC values, respectively. The micellar systems were titrated with increasing amounts of PhTD-ClB dissolved in methanol (5, 10, 30, 50, and 60 µL) to obtain concentrations of 4.97 × 10^−6^ M, 9.9 × 10^−6^ M, 2.95 × 10^−5^ M, 4.89 × 10^−5^ M, 5.84 × 10^−5^ M, respectively. As shown in Figure 6, similarly to Figure 1, a characteristic absorption band was observed for PhTD-ClB in the micellar system, with a maximum at around 330 nm for <CMC and for CMC (Panel A and B) and around 345 nm for 2 CMC (Panel C). An interesting effect can be observed here, namely a shift in the main absorption maximum towards shorter wavelengths with increasing PhTD-ClB concentration in the micellar system. For <CMC and CMC concentrations, the effect is very strong, while for 2 CMC it is observed at the highest thiadiazole concentrations. This indicates a visible solvatochromic shift towards shorter wavelengths with increasing PhTD-ClB concentration in each micellar system. This band, in accordance with well-documented literature data [71], is characteristic of the π→π* electronic transition in the 1,3,4-thiadiazole molecule, as described above.

Depending on the different PhTD-ClB concentrations, significant changes in the shape of the recorded spectra can be observed, much more visible than in Figure 1. In all presented absorption spectra, an increase in the band intensity is observed on the longer wavelength side, with a maximum at around 360 nm (in panels B and C) and 380 nm (in panel A). A broad shoulder appears on the longwave side of the measured absorption spectra. In Panels B and C, a clear shift towards the shortwave side is also characteristic. This broadening on the longwave side, consistent with M. Kasha’s excitonic splitting theory, suggests the presence of non-monomeric forms of PhTD-ClB (such as dimers or N-type aggregates) in the analyzed samples. For the spectra shown in Panel A (<CMC TX-100), slight scattering is observed, although the aggregation effect is very pronounced, similarly to Panel B. In contrast, in Panel C, distinct aggregation bands are visible on the long-wavelength side, with a maximum around 365 nm, and there is much less light scattering. This is a consequence of the formation of micelles, which solubilize the aggregated forms of thiadiazole without causing strong light scattering.

It is worth noting that a very strong shift in the absorption band from 345 to 330 nm, visible in Panels B and C, occurs after the second addition of the compound (change from 4.97 × 10^−6^ M to 9.9 × 10^−6^ M) to the medium. After the last addition (5.84 × 10^−5^ M) of the compound, the absorption band shifts even more strongly. It is somewhat surprising that aggregation effects in the absorption spectra are more pronounced at the lowest detergent concentration and in its CMC. This may be due to the aggregation of PhTD-ClB in the PBS buffer, enhanced by the presence of a small amount of detergent. Nevertheless, the micellar system clearly influences the induction of aggregation effects by effectively modifying the hydrophobicity of the environment. Analogous results for the second surfactant (NADC) are presented in the Supporting Materials in Figures S10–S12.

**Figure 7:**
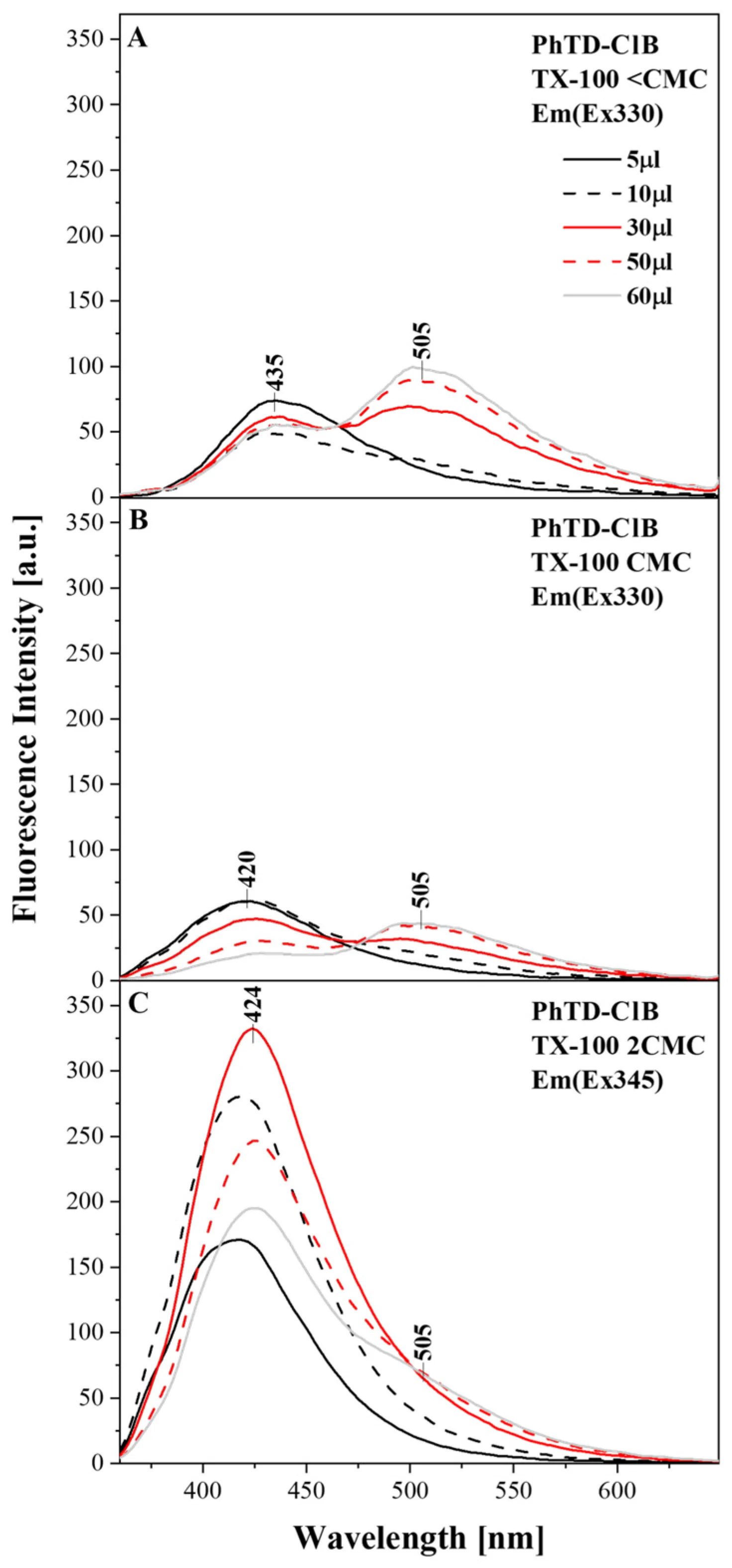
Fluorescence emission spectra of PhTD-ClB at excitation wavelengths of 345 and 330 nm in a micellar system with concentrations <CMC (panel (**A**)), CMC (panel (**B**)) and 2 CMC (panel (**C**)). The PhTD-ClB concentrations are: 4.97 × 10^−6^ M, 9.9 × 10^−6^ M, 2.95 × 10^−5^ M, 4.89 × 10^−5^ M, 5.84 × 10^−5^ M.

Particularly noteworthy are the results presented in Figure 7, where the fluorescence emission spectra corresponding to the UV-Vis absorption spectra are presented (from Figure 6). In this case, the excitation wavelength for all samples corresponded to the location of the main maximum in the absorption spectrum. As shown in panels A–C, depending on the concentration of PhTD-ClB in the micellar system formed from TX-100, as well as the amount of detergent used, a clear dual fluorescence effect is observed.

Panel A refers to the first stage of the experiment, i.e., the system before reaching the CMC, and shows that dual fluorescence appears practically immediately following the addition of the second portion of the compound (9.9 × 10^−6^ M), with maxima at 435 and 505 nm—similarly to solvents of lower polarity (e.g., CHCl_3_) or in water (Figure 2). The emission band with a maximum at around 400 nm, characteristic for polar solvents, disappears (Figure 2). The maximum at ~400 nm is associated with π–π* transitions of resorcinol–thiadiazole and CT in the substituent system of the molecule→resorcinol–thiadiazole within the cis-enol form, as described in our previous publications for similar thiadiazole systems [72].

As the amount of compound added increases, the long-wave emission band intensifies, reaching a maximum at around 505 nm. These results are consistent with our previous studies on similar molecules [73]. Interestingly, it can be stated that in the system below CMC (<CMC), the surfactant concentration is quite low and insufficient to form micellar systems; however, its small amount effectively forces aggregation effects, due to which the molecules of the selected thiadiazole start to aggregate with each other, which is revealed in the emission spectra as dual fluorescence [74]. The relatively high quantum yield of the analyzed 1,3,4-thiadiazole is also clearly visible here. Despite aggregation effects, increasing the compound concentration in the medium enhances the emission band at 505 nm, clearly associated with the cis-enol→keto* transition. Therefore, the enhancement of the long-wavelength emission under these conditions is consistent with aggregation-facilitated ESIPT and AIE-like behavior [25].

Interesting effects can also be observed in Panel B, where, depending on the amount of thiadiazole added, at a surfactant concentration equal to the CMC enabling micelle formation, two emission bands characteristic of the ESIPT effect can be observed. As shown in Figure 6B, with an added amount of PhTD-ClB of 10 µL (9.9 × 10^−6^ M), a long-wave band with a maximum at around 500 nm begins to appear, and with increasing concentration of 1,3,4-thiadiazole used, the effect intensifies. However, this effect is not as intense as in Panel A of this figure. The shift in the short-wavelength emission band from approximately 435 to 420 nm indicates a change in the local environment and/or molecular organization of PhTD-ClB, but does not by itself distinguish between monomeric and aggregated species. The band centered near 430 nm may contain contributions from PhTD-ClB molecules located in a less polar micellar microenvironment and from associated species; its assignment specifically to dimers cannot be made on the basis of the steady-state spectra alone. In this case, it can be seen that at a sufficiently low concentration of thiadiazole and a sufficient amount of detergent to form micelles, some of the added PhTD-ClB molecules may remain in the monomeric form. As the compound concentration increases and the surfactant concentration remains unchanged, a decrease in the intensity of the short-wavelength band (420 nm) is observed, although it is still present, and at the same time an emission band with a maximum at 505 nm appears, similarly to panel A. Both literature data and the results of our previous studies of this molecule suggest that this band is characteristic of the ESIPT effect occurring in PhTD-ClB and is related to the emission of the keto* tautomer [75,76]. The spectral changes indicate that the extent of molecular association depends on both the PhTD-ClB concentration and the surfactant concentration relative to the CMC. It is worth noting that, despite significant aggregation, no decrease in the intensity of the emission spectra was observed.

Panel C of Figure 7 presents the fluorescence emission spectra at a fixed amount of TX-100 corresponding to the concentration of 2 × CMC, while the concentrations of PhTD-ClB were the same as in the previous experiments (Figure 6). However, contrary to our previous studies, at the concentration of 2 CMC we observed practically a single fluorescence emission spectrum with increasing compound. Only after the final addition of PhTD-ClB, corresponding to 60 μL of the stock solution and a final concentration of 5.84 × 10^−5^ M, did a weak long-wavelength emission band centered near 505 nm become apparent. It should be emphasized that for PhTD-ClB we observe completely different results than in our previous studies of other analogs from this group of 1,3,4-thiadiazoles. It is clearly visible that aggregation effects, although present across the entire range of detergent concentrations, have the greatest impact on the ESIPT effect observed at <CMC and CMC concentrations. At a detergent concentration of 2 CMC, which exhibits a higher number of micelles, PhTD-ClB molecules are likely to remain in their monomeric form. Only at higher amounts of PhTD-ClB do they begin to aggregate and a pronounced ESIPT effect begins to occur.

As demonstrated in the experiment, the observed fluorescence effects depend on both the surfactant concentration and the PhTD-ClB concentration in the micellar system. The results clearly indicate the influence of molecular aggregation effects, which we observe here in the model biophysical system of micelles. It should also be emphasized that in this case, the appropriate detergent concentration (CMC and 2 × CMC) causes solubilization of PhTD-ClB aggregates, which is manifested by the appearance of a maximum at around 410 nm. The lower the thiadiazole concentration, the higher the intensity of the band, with a maximum at around 420 nm.

Overall, the results show that the ESIPT-associated dual emission of PhTD-ClB can be modulated by changing the surfactant concentration and, consequently, the hydrophobic organization of the medium. PhTD-ClB was initially dissolved in a polar medium, in which only the emission of the excited enol tautomer of this compound was consistently observed (Figure 2). Analogous results for the second analog used in this study in TX-100, as well as for both of these analogs in the second, clinically used detergent, NaDC, are presented in the Supporting Materials in Figures S10–S16. We also observed analogous effects of the dependence of ESIPT on the amount of both the detergent system and the compound itself. These results demonstrate that ESIPT-related emission can be modulated in surfactant-based carrier systems, thereby expanding the potential applications of these compounds. However, it should be emphasized that the selected two analogs from the 1,3,4-thiadiazole group behave quite differently from our previous results for other compounds from this group [68], because we clearly observe that at higher detergent concentrations, these compounds monomerize. At higher surfactant concentrations, a greater concentration of the thiadiazole is required to promote intermolecular association and the appearance of ESIPT-associated dual emission.

**Figure 8:**
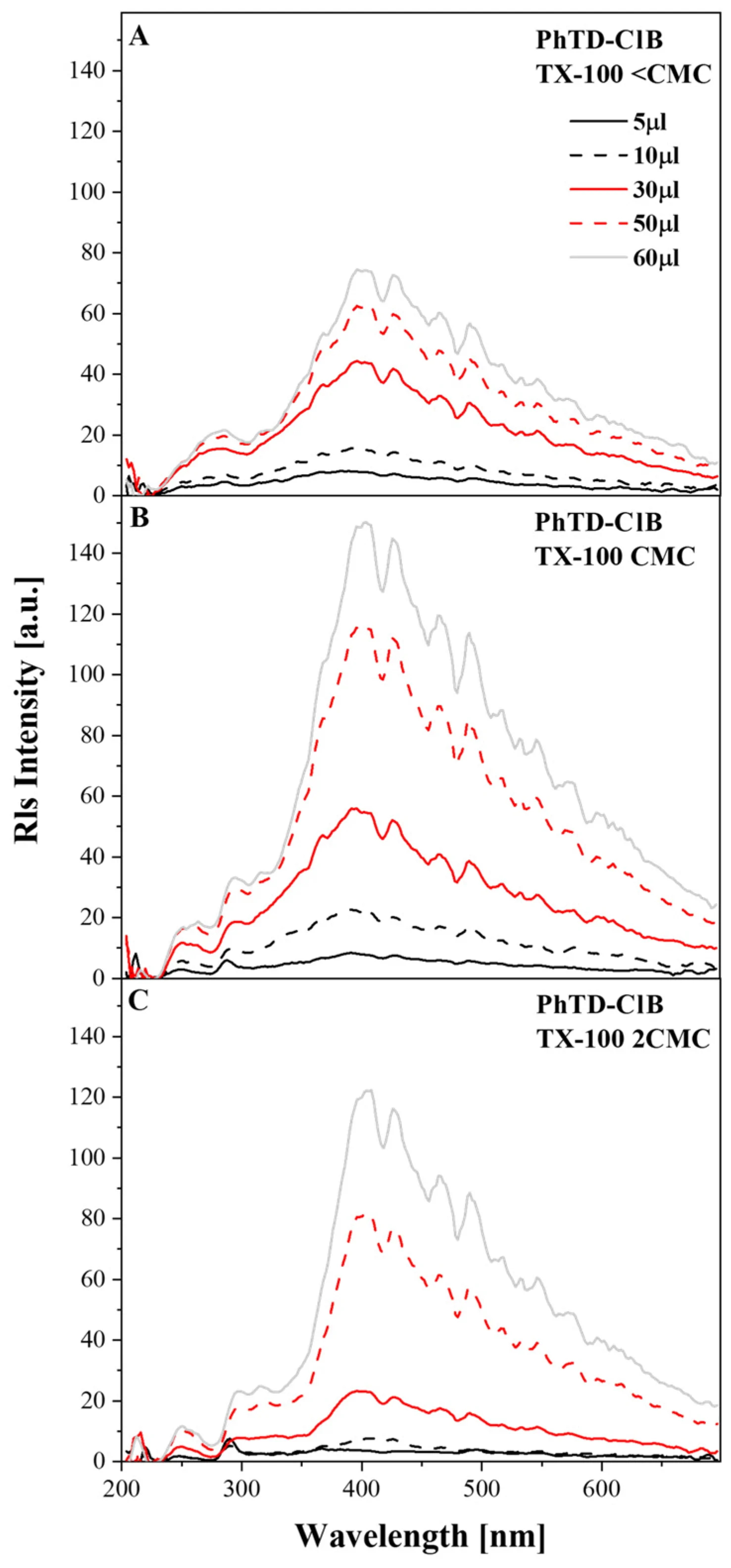
Resonance light scattering spectra of PhTD-ClB in the micellar system at <CMC (panel (**A**)), CMC (panel (**B**)) and 2 CMC (panel (**C**)). The compound concentrations are: 4.97 × 10^−6^ M, 9.9 × 10^−6^ M, 2.95 × 10^−5^ M, 4.89 × 10^−5^ M, 5.84 × 10^−5^ M.

The results presented in Figure 8 correspond to those presented in Figure 7, which illustrate the resonance light scattering (RLS) spectra obtained for the PhTD-ClB compound in a micellar system. According to the literature data, the occurrence of RLS bands should be primarily associated with the presence of various aggregated forms in the studied system, such as a dimer or larger N-aggregated systems [77]. As can be seen in Figure 8, Panels A-C present results corresponding in composition and concentration to those in Figure 6 and Figure 7. As can be seen in Panel A, for concentrations <CMC, RLS bands appear already after the third addition of PhTD-ClB (compound concentration was 2.95 × 10^−5^ M). In Panel B, a similar trend was observed, whereas in Panel C a pronounced RLS signal appeared only at the highest PhTD-ClB concentrations. This behavior is consistent with a lower contribution of large scattering species at low compound concentrations in the 2 CMC system, although it does not unequivocally demonstrate complete monomerization. It should also be noted that the relative intensity of the RLS bands is higher in Panels B and C than in Panel A (<CMC). This can be explained by comparing the size of the potential PhTD-ClB dimer with the monomeric form of the compound present in the TX-100 micelle. The higher RLS intensity is consistent with an increased contribution of larger scattering species, whose scattering cross-section is greater than that of the monomeric form. Additionally, the oscillatory structure of the obtained synchronous spectra also indicates that different aggregation forms occur in the studied systems.

**Figure 9:**
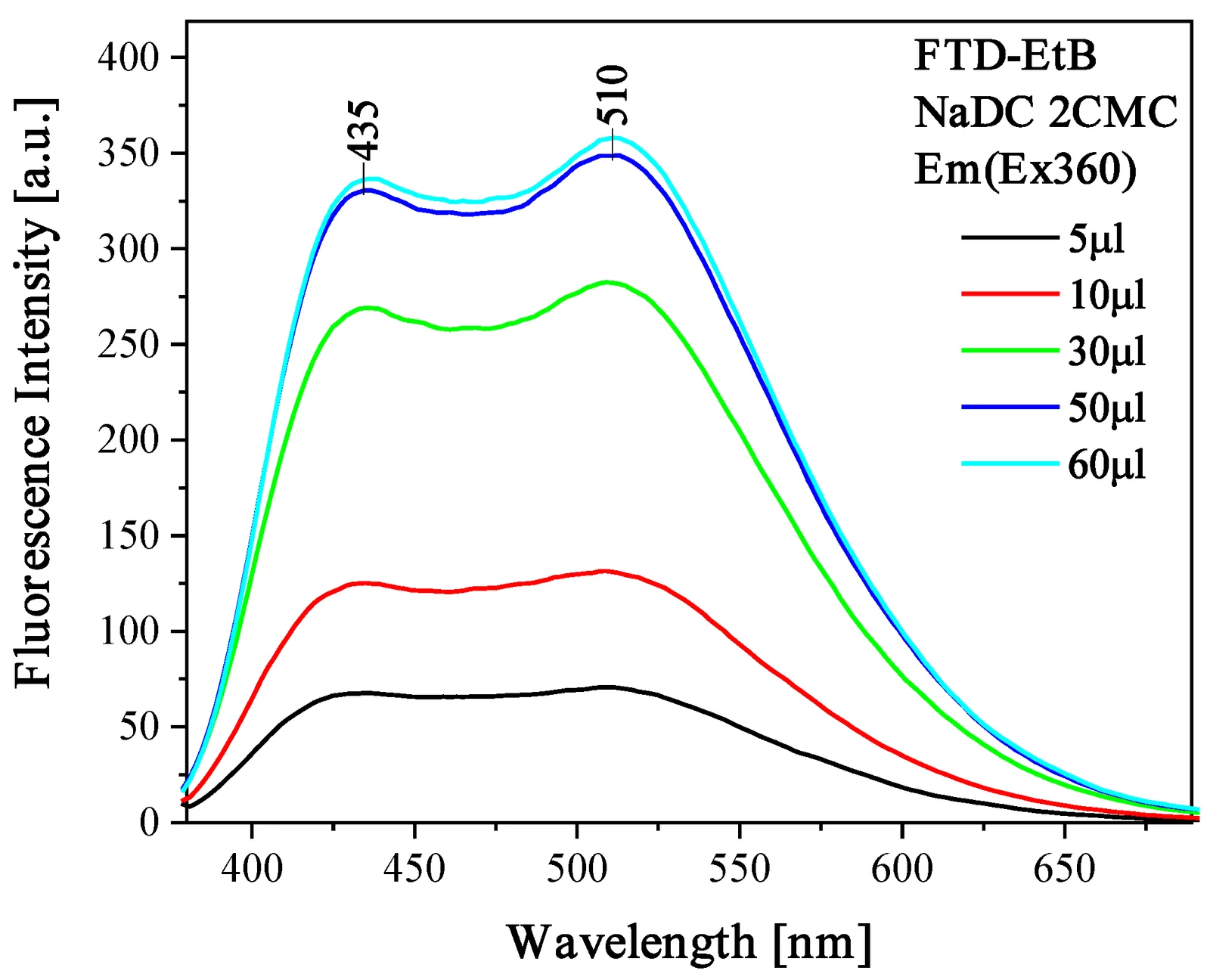
Fluorescence emission spectra of FTD-EtB at 360 nm excitation wavelength in a micellar system with a concentration of 2 CMC. The compound concentrations are respectively 4.05 × 10^−6^ M, 8.06 × 10^−6^ M, 2.4 × 10^−5^ M, 3.98 × 10^−5^ M, and 4.75 × 10^−5^ M.

To complement the more extensive micellar studies performed for PhTD-ClB, the spectroscopic behavior of FTD-EtB was also investigated in the 2 CMC micellar system. Figure 9 presents the fluorescence emission spectra of FTD-EtB recorded upon excitation at 360 nm at increasing compound concentrations. The spectra show a systematic concentration-dependent fluorescence response of FTD-EtB in the organized micellar environment, demonstrating that its excited-state behavior is sensitive to the presence of the micellar phase. The corresponding absorption, fluorescence excitation, and RLS spectra recorded for FTD-EtB under the same 2 CMC conditions are provided in the Supplementary Materials (Figures S14–S16).

### 2.6. Antifungal Activities

**Figure 10:**
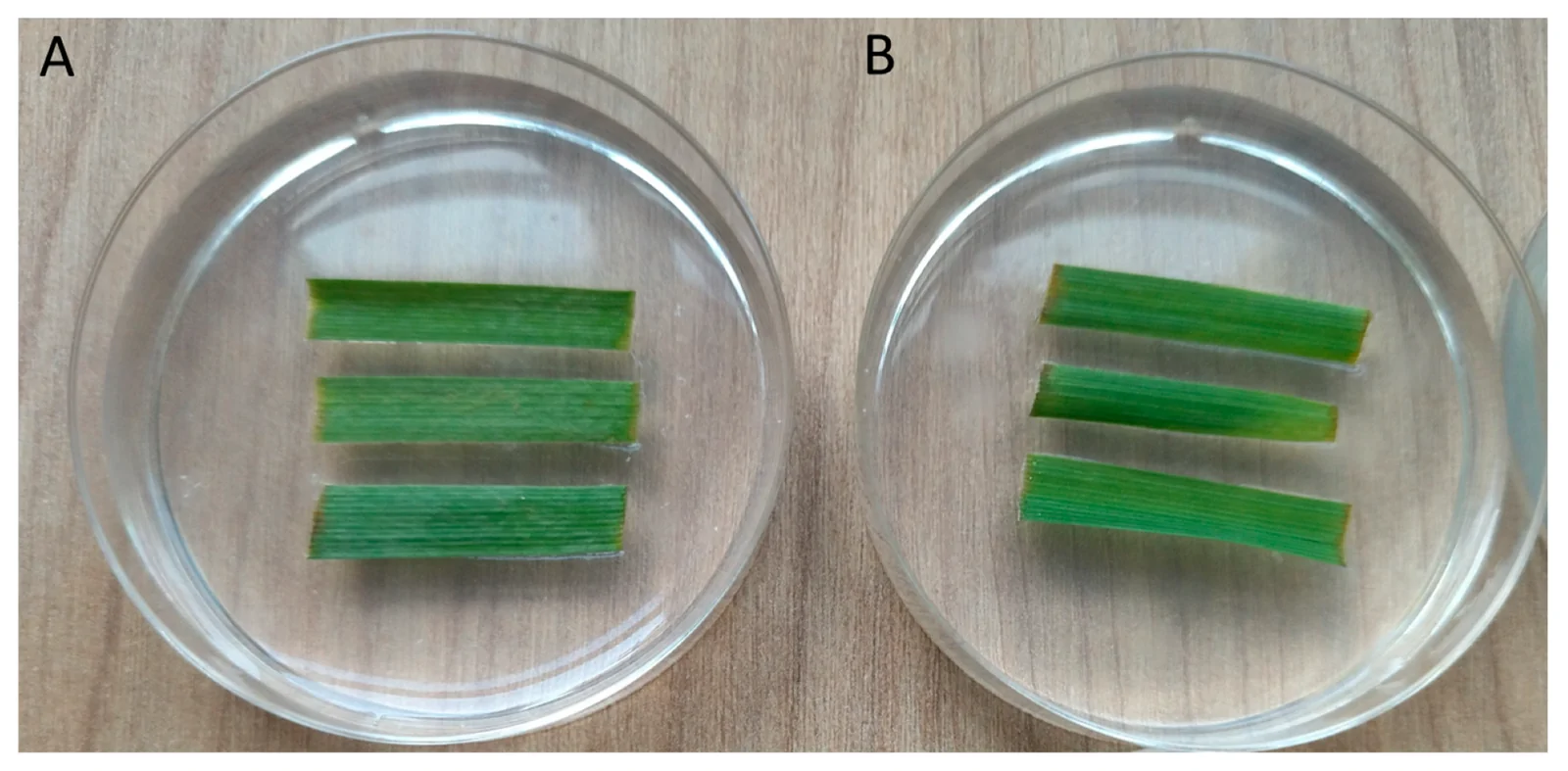
Example result of host-pathogen test conducted in vitro on leaf fragments in the presence of tested compounds. (**A**) Leaves of a susceptible oat variety (Fuchs) placed in a medium with 5 µg∙mL^−1^ PhTD-ClB inoculated with *B. graminis* f.sp. *avenae* spores. (**B**) Leaves of a susceptible oat variety (Fuchs) placed in a medium with 5 µg∙mL^−1^ FTD-EtB inoculated with *B. graminis* f.sp. *avenae*.

The biological activity of 1,3,4-thiadiazole derivatives is very broad. Besides their key importance in medicine and pharmacy, these compounds have also found applications in agriculture and horticulture as potential plant protection agents, particularly in the control of fungal pathogens. To date, their effectiveness has been demonstrated against many pathogenic organisms, such as *Colletotrichum orbiculare*, *Bipolaris maydis*, *Alternaria solani*, *Fusarium oxysporum* and *Botrytis cinerea* [21,78,79]. Biotrophic pathogens are particularly important in cereal crops as they significantly reduce the quality and yield of crops [80,81]. In our work, we attempted to check the activity of 1,3,4-thiadiazole derivatives against the most important biotrophic fungal pathogens that attack wheat and oats, namely against *Blumeria graminis* f.sp. *avenae*, *Blumeria graminis* f.sp. *tritici*, *Puccinia coronata* f.sp. *avenae* and *Puccinia recondita* f.sp. *tritici*. Fungi of the genus *Blumeria* cause powdery mildew in cereals and grasses. As obligate biotrophic pathogens, they may cause substantial yield losses [82]. Fungi of the genus *Puccinia* cause leaf diseases called rusts. These pathogens cause significant economic losses in cereal crops [83]. Protecting cereal crops from pathogen attack relies on two strategies. On the one hand, it involves introducing varieties with genetically determined resistance. However, this strategy requires introducing a group of resistance genes with varying levels of effectiveness, which reduces the risk of pathogen populations overcoming their resistance [84]. The second strategy, in turn, relies on the use of chemical plant protection products. Agrochemicals currently used to protect plants from pathogens have different modes of action. These products operate primarily through contact, eliminating pathogens on the plant surface, and systemically, penetrating plant tissues [85]. Many agrochemicals exhibit phytotoxicity, which is a negative effect of plant protection products on plants, leading to inhibition of their growth and development [86]. Therefore, it is necessary to seek new solutions that can be used to control fungal pathogens. Our study assessed the activity of selected 1,3,4-thiadiazole derivatives against biotrophic cereal pathogens. Of the compounds tested, PhTD-ClB showed no activity against *Blumeria* and *Puccinia* species at any of the tested concentrations. The compound FTD-EtB behaved differently, significantly limiting the growth and development of both groups of pathogens across the entire range of concentrations used in the experiment (Table 4, Figure 10).

These results identify FTD-EtB, but not PhTD-ClB, as a candidate for further investigation as an antifungal agent against biotrophic cereal pathogens and as a potential lead structure for the development of more sustainable plant-protection strategies.

## 3. Materials and Method

### 3.1. Materials

**Synthesis**:

PhTD-ClB and FTD-EtB were obtained from the previously established synthetic series developed at the Department of Chemistry, University of Life Sciences in Lublin. Both compounds are known chemical entities whose synthesis and analytical characterization have been reported previously. PhTD-ClB and FTD-EtB were characterized by ^1^H/^13^C NMR spectroscopy, EI-MS, IR spectroscopy and elemental analysis [87,88]

**PhTD-ClB and FTD-EtB in solvents**: To prepare the stock solutions, 1 mg of each compound was dissolved in 1 mL of the selected solvent (methanol, ethanol, propan-2-ol, butan-1-ol, glycerol, water, acetonitrile, ethyl acetate, THF, chloroform, DMF, DMSO). All the solvents used in this study were of analytical grade. Aliquots of the stock solutions were diluted with the corresponding solvents to obtain absorbance values suitable for spectroscopic measurements. For spectroscopic measurements in pure solvents, the final concentration was maintained constant for each compound and was 2.95 × 10^−5^ M for PhTD-ClB and 2.40 × 10^−5^ M for FTD-EtB. Because the resulting concentrations were not strictly identical in all solvents, the normalized absorption spectra presented in Figure 1 were used for qualitative comparison of spectral positions and band shapes rather than for quantitative comparison of absorbance intensities between solvents.

**Micellar system**: Micellar systems: PhTD-ClB (MW = 354.21 g × mol^−1^) and FTD-EtB (MW = 288.32 g × mol^−1^) were separately dissolved in methanol to prepare the respective stock solutions. The volume of the PhTD-ClB and FTD-EtB stock solution added to 3 mL PBS (phosphate-buffered saline—10 mM, pH 7.4) was set at 5, 10, 30, 50 and 60 μL, thus resulting in respective thiadiazols concentrations of 4.97 × 10^−6^ M, 9.9 × 10^−6^ M, 2.95 × 10^−5^ M, 4.89 × 10^−5^ M, 5.84 × 10^−5^ M for PhTD-ClB and 4.05 × 10^−6^ M, 8.06 × 10^−6^ M, 2.4 × 10^−5^ M, 3.98 × 10^−5^ M, 4.75 × 10^−5^ M for FTD-EtB. The micellar systems were prepared by mixing appropriate amounts of the surfactant and buffer (PBS). A stock solution of Triton X-100 (28 mM) was prepared by dissolving 100 μL of the Triton X-100 reagent (Sigma Aldrich, Poznań, Poland) in 6 mL of water. Triton X-100 concentrations were adjusted to 0.09 mM (below CMC), 0.22 mM (CMC), and 0.44 mM (2 × CMC). Sodium deoxycholate solution was prepared at the following concentrations: 3 mM (below CMC), 6 mM (CMC), and 12 mM (2 × CMC).

### 3.2. Methods

#### 3.2.1. Electronic Absorption and Fluorescence Spectra

The electronic absorption spectra of selected 1,3,4-thiadiazole derivatives were recorded using a Cary 300 Bio double-beam UV-Vis spectrophotometer (Varian, Warsaw, Poland), equipped with a thermostatted sample holder featuring a 6 × 6 multi-cell Peltier block. Temperature control was maintained at 22 °C via a thermocouple probe (Cary Series II, Varian) placed directly in the sample solution.

Fluorescence excitation, emission, and synchronous spectra (resonance light scattering, RLS) were acquired using a Cary Eclipse spectrofluorometer (Varian). All fluorescence measurements were conducted at a constant temperature of 22 °C, with a spectral resolution of 0.5 nm. The spectra were corrected for both the excitation source and photomultiplier spectral response characteristics.

Resonance light scattering (RLS) measurements were performed following a previously reported protocol, utilizing synchronous scanning of the excitation and emission monochromators (zero wavelength offset), with a spectral resolution of 1.5 nm.

All spectral data were processed and analyzed using Grams/AI 8.0 software (Thermo Electron Corporation, Waltham, MA, USA).

#### 3.2.2. Time-Correlated Single Photon Counting

Time-correlated single photon counting (TCSPC) measurements were performed using a FluoroCube fluorimeter (Horiba, Loos, France). The samples were excited with a pulsed NanoLED diode at 372 nm (pulse duration of 150 ps) operated with 1 MHz repetition. To avoid pulse pile-up, the power of the pulses was adjusted to an appropriate level using a neutral gradient filter. Fluorescence emission was recorded using a picosecond detector TBX-04 (IBH, JobinYvon, UK). Data acquisition was performed using DataStation software (version 2.1.6), while data analysis was carried out using DAT6 software (version 6.1.99). All fluorescence decays were measured in a 10 × 10 mm quartz cuvette using an emission cut-off filter transmitting wavelengths above 408 nm. The excitation profiles required for the deconvolution analysis were measured without the emitter filters using a light scattering cuvette. The compounds were dissolved in DMSO or PBS directly before the measurements. Each fluorescence decay was analyzed with a multiexponential model given by the following equation:

(1) $$I_{t}=\sum_{i}\alpha_{i}\exp\left(-\frac{t}{\tau_{i}}\right)$$

where α_ii_ and τ_ii_ are the pre-exponential factor and the decay time of component *i*, respectively.

Best-fit parameters were obtained by minimization of the reduced χ^2^ value, as well as residual distribution of the experimental data. The fractional contribution (*f_i_*) of each decay time and the average lifetime of fluorescence decay (<τ>) were calculated according to the following equations:

(2) $$f_{i}=\frac{\alpha_{i}\tau_{i}}{\sum_{j}\alpha_{j}\tau_{j}}$$

(3) $$\left\langle \left.\tau \right\rangle \right.=\sum_{i}f_{i}\tau_{i}$$

#### 3.2.3. Fluorescence Quantum Yields

The fluorescence quantum yields of PhTD-ClB and FTD-EtB were determined relative to 7-diethylamino-4-methylcoumarin (Coumarin 1) used as the fluorescence standard. Coumarin 1 in ethanol, with a reported fluorescence quantum yield of *Φ*_F_ = 0.73, was used as the reference [25]. The final fluorescence quantum yield values were calculated based on Equation (4):

(4) $$\phi_{F(X)}=\phi_{R\left(EtOH\right)}\left(\frac{\lambda_{exR\left(EtOH\right)}}{\lambda_{exX}}\right)\left(\frac{I_{X}}{I_{R(EtOH)}}\right)\left(\frac{\eta_{X}^{2}}{\eta_{R(EtOH)}^{2}}\right)$$

where the subscript X denotes selected compounds in various solvents and mixed systems and the subscript R(EtOH) denotes coumarin1 in EtOH solution, λ_ex_ is the value of absorbance at the excitation wavelength, I is the area under the emission curve, and η is the refractive index of the solvent.

#### 3.2.4. Methodology and Calculation of Dipole Moments

Changes in molecular dipole moment upon excitation can be estimated using solvatochromic methods based on the relationship between the positions of the absorption and fluorescence maxima and solvent-polarity parameters, including the dielectric constant (ε) and refractive index (n). In this paper the ground and excited dipole moment were calculated based on two methods: Bakhshiev and Kawski–Chamma–Viallet, as well as microscopic solvent parameters ($E_{T}^{N}$).

**Method 1**

The dipole moment of molecules *4-chloro-6-{5-[(3-chlorophenyl)amino]-1,3,4-thiadiazol-2-yl}benzene-1,3-diol* (PhTD-ClB) and *4-ethyl-6-[5-(furan-2-yl)-1,3,4-thiadiazol-2-yl]benzene-1,3-diol* (FTD-EtB) was determined by the Bakhshiev and Kawski–Chamma–Viallet methods [89,90]. The change in dipole moment can be estimated using two following relations (5) and (6):

**Bakhshiev**

(5) $$v_{a}-v_{f}=S_{1}F_{1}\left(\varepsilon ,n\right)+const$$

**Bilot-Kawski**

(6) $$\frac{v_{a}+v_{f}}{2}=S_{2}F_{2}\left(\varepsilon ,n\right)+const$$

where $v_{a}$ and $v_{f}$ are the maxima of absorbance and fluorescence in cm^−1^. Functions F_1_ and F_2_ (7, 8) depend on the dielectric constant (e) and refractive effect (n) of the solvent. They can be presented as the two following expressions:

(7) $$F_{1}\left(\varepsilon ,n\right)=\frac{2n^{2}+1}{n^{2}+2}\left(\frac{\varepsilon -1}{\varepsilon +2}-\frac{n^{2}-1}{n^{2}+2}\right)$$

(8) $$F_{2}\left(\varepsilon ,n\right)=\frac{2n^{2}+1}{{2(n}^{2}+2)}\left(\frac{\varepsilon -1}{\varepsilon +2}-\frac{n^{2}-1}{n^{2}+2}\right)+\frac{\left(n^{4}-1\right)}{2(n^{2}+2{)}^{2}}$$

Assuming that the symmetry of the investigated solute molecule remains unchanged upon electronic transition, and the ground and excited-state dipole moments are parallel, we obtain the following expressions (Equations (9) and (10)):

(9) $$\mu_{g}=\left|\frac{S_{1}+S_{2}}{2}\right|{\left(\frac{hca_{0}^{3}}{2S_{1}}\right)}^{1/2}$$

(10) $$\mu_{e}=\left|\frac{S_{1}-S_{2}}{2}\right|{\left(\frac{hca_{0}^{3}}{2S_{1}}\right)}^{1/2}$$

where $\mu_{g}$ and $\mu_{e}$ are the ground and excited state dipole moments, h is Planck’s constant, c is the velocity of light, and a_0_ is the Onsager cavity radius as calculated from the Van der Waals volumes ($V=\frac{3}{4}\pi a_{0}^{3})$ according to Edward’s method [59]. The ratio of excited state dipole moment to the ground state dipole moment can be expressed as (Equation (11)):

(11) $$\frac{\mu_{e}}{\mu_{g}}=\left|\frac{S_{1}-S_{2}}{S_{1}+S_{2}}\right|$$

Slopes S_1_ and S_2_ have been determined by plotting the Stokes shifts and $\frac{v_{a}+v_{f}}{2}$ against the bulk solvent polarity functions $F_{1}(\varepsilon ,n)$ and $F_{2}(\varepsilon ,n)$ respectively for different solvents. Where $\nu_{a}$ is absorption maximum in cm^−1^ and $\nu_{f}$ is fluorescence maximum in cm^−1^.

Generally, when dipole moments $\mu_{e}$ and $\mu_{g}$ are not parallel to each other and form an angle ϕ, the use of (12) and (13) leads to [91]:

(12) $$\mu_{e}=(\mu_{g}^{2}+\frac{1}{2}S_{2}hca_{0}^{3}{)}^{1/2}$$

and an angle ϕ can be calculated using Equation (9):

(13) $$cos\phi =\frac{1}{2\mu_{g}\mu_{e}}\cdot [\left(\mu_{g}^{2}+\mu_{e}^{2}\right)-\frac{S_{1}}{S_{2}}\left(\mu_{e}^{2}-\mu_{g}^{2}\right)]$$

The change in dipole moment can be determined as (Equation (14))

(14) $$∆\mu =\mu_{e}-\mu_{g}$$

**Method 2**

The effect of polarization and hydrogen bonding with solvents can be expressed by the function E_T_(30), which is useful to measure solvent polarity using the solvatochromic method with betaine dye as a probe solute [92]. The empirical Dimroth–Reichardt polarity parameter provides a complementary description of solvent effects because, unlike polarity functions based solely on the dielectric constant and refractive index, it is sensitive to microscopic solvent polarity and specific solute–solvent interactions, including hydrogen bonding. The change in dipole moment can be expressed by the following equation (Equation (15)):

(15) $$v_{a}-v_{f}=m\cdot E_{T}^{N}+const$$

where microscopic solvent polarity $\left(E_{T}^{N}\right)$ is a normalized value of E_T_(30) and is defined using water ($E_{T}^{N}=1)$ and tetramethylsilane ($E_{T}^{N}=0)$ as an extreme reference solvent with the following equation (Equation (16)):

(16) $$E_{N}^{T}=\frac{E_{T}\left(solvent\right)-E_{T}(TMS)}{E_{T}\left(water\right)-E_{T}(TMS)}=\frac{E_{T}\left(solvent\right)-30.7}{32.4}$$

The change in dipole moment can be determined as (Equation (17))

(17) $$∆\mu =\mu_{e}-\mu_{g}=\sqrt{\frac{81m}{(6.2/a_{0}{)}^{3}11307.6}}$$

where m is the slope of the linear plot of $E_{T}^{N}$ versus Stokes shift. The values of E_T_(30) and $E_{T}^{N}$ for the solvent are presented in Table S2.

#### 3.2.5. Rate Constants of Radiative (*k_r_*) and Non-Radiative (*k_nr_*) Intramolecular Processes

Quantum yield (*Φ*) is defined as the ratio of the number of photons emitted to the number of photons absorbed and linked with radiative and non-radiative process rate constants via Equation (18) [93]:

(18) $$\phi =\frac{k_{r}}{k_{nr}+k_{r}}=\frac{number\;of\;photon\;emitted}{number\;of\;photon\;absorbed}$$

where *k_r_* is the rate constant for the radiative process, while *k_nr_* is the rate constant for non-radiative process. The measured lifetime of fluorescence (τ) can be related to the rate constants for radiative and non-radiative decay *k_r_* and *k_nr_*, as presented in Equation (19):

(19) $$\tau =\frac{1}{k_{r}+k_{nr}}$$

Consequently, the basic photophysical equations which correlate the quantum yield and lifetime of fluorescence of the molecule with rate constants of radiative and non-radiative processes can be defined as follows [94]:

(20) $$k_{r}=\frac{\phi_{F}}{\tau }$$

(21) $$k_{nr}=\frac{1-\phi_{F}}{\tau }$$

where in our case $\phi_{F}$ and τ are, respectively, the fluorescence quantum yield and the fluorescence lifetime measured for the thiadiazole solution.

#### 3.2.6. Antifungal Activities

Antifungal activities were tested in vitro against four of the most damaging pathogens affecting oat and wheat: *Blumeria graminis* f.sp. *avenae*, *Blumeria graminis* f.sp. *tritici*, *Puccinia coronata* f.sp. *avenae* and *Puccinia recondita* f.sp. *tritici*. The target compounds were dissolved in DMSO to obtain stock solutions at a concentration of 100 µg/mL and were subsequently tested at final concentrations of 1, 2, 3, 4, 5, and 10 µg/mL. Appropriate volumes of the stock solutions were added to 10 mL of agar medium (6 g/L) to obtain the required compound concentrations. For each tested concentration, a solvent-matched control was prepared by adding the same volume of DMSO to the agar medium as that introduced with the corresponding compound stock solution. Leaves of the Fuchs (*A. sativa*) and Błyskawica (*T. aestivum*) cultivars susceptible to analyzed pathogens were placed on the Petri dishes with the agar medium and the analyzed compounds. Control leaf fragments were placed on the corresponding solvent-matched control media prepared as described above. Because the relatively low concentration of the DMSO stock solution required comparatively large solvent volumes at the highest compound concentrations, the resulting DMSO content represents a limitation of the present biological assay; however, its contribution was controlled by the use of concentration-matched DMSO controls. Petri dishes with leaf fragments were inoculated with pathogen spores according to the host-pathogen test methodology described by Hsam et al. [93]. After 10 days, the infection of the leaves was assessed on a five-point scale presented in Table 5 [94].

The results of the percentage disease control were calculated compared with the control plants, wherein 100 were rated as complete disease control and 0 as no disease control [95]. Each compound–pathogen–concentration combination was tested using four independent leaf fragments, and the entire experiment was repeated three times. The values presented in Table 4 represent the modal infection score obtained across the biological replicates.

## 4. Conclusions

The presented comprehensive spectroscopic studies performed in environments of variable polarity and hydrophobicity, supported by dipole moment calculations and molecular distance calculations using M. Kasha’s excitonic splitting theory, provide evidence that the dual fluorescence phenomenon observed in the spectra of PhTD-ClB and FTD-EtB molecules is related to the process of excited-state intramolecular proton transfer (ESIPT) of the cis-enol*→ keto* type. The higher-energy emission band in the fluorescence spectrum originates from the excited enol form, occurring mainly in polar solvents, while the lower-energy band originates from the excited keto form of the studied analogs. In solvent mixtures where varying hydrophobicity favors interactions between the studied 1,3,4-thiadiazole molecules, the dual fluorescence effects were strongly modified by aggregation phenomena associated with aggregation-induced emission (AIE). In micellar systems formed by TX-100 and NaDC, molecular aggregation promoted the appearance or enhancement of dual fluorescence. Molecular association and incorporation into micellar environments may partially shield the intramolecular hydrogen-bonding site from direct solvent interactions, thereby favoring the ESIPT pathway and the associated long-wavelength emission. However, with increasing detergent concentration, increased monomerization of selected analogs was observed. Lower overall fluorescence quantum yields were observed under conditions in which the keto-related long-wavelength emission was prominent, whereas higher yields accompanied predominantly enol-related emission.

Among the two derivatives, only FTD-EtB exhibited pronounced antifungal activity, reducing visible disease symptoms under the experimental conditions across the investigated concentration range. PhTD-ClB showed no detectable antifungal effect under the same conditions.

## Figures and Tables

**Table 1:** Measurements (at the excitation wavelength λ_ex_) of PhTD-ClB and FTD-EtB surface area I under the emission curve and fluorescence quantum yield *Φ*_F_ in pure solvents.

Nr	Solvent	PhTD-ClB	FTD-EtB	PhTD-ClB	FTD-EtB
		I	I	*Φ*_F_	*Φ*_F_
1	MeOH	21,009	8355	0.24	0.1
2	EtOH	15,963	7276	0.19	0.09
3	Propan-1-ol	10,984	5835	0.14	0.07
4	Butan-1-ol	8715	6155	0.11	0.08
5	ACN	2018	7834	0.02	0.09
6	Ethyl acetate	3702	2132	0.05	0.03
7	THF	8611	7597	0.11	0.1
8	Chloroform	1737	1657	0.02	0.02
9	DMF	25,669	12,573	0.34	0.17
10	DMSO	34,555	18,394	0.49	0.26
11	Glycerol	39,547	14,539	0.55	0.2

**Table 2:** Measurements of PhTD-ClB and FTD-EtB fluorescence average lifetimes <τ> and fluorescence quantum yield *Φ*_F_ in pure solvents. Calculations of PhTD-ClB and FTD-EtB rate constants of radiative (*k_r_*) and non-radiative (*k_nr_*) intramolecular processes in pure solvents.

PhTD-ClB						
Solvent	<τ>	*Φ*_F_	*k_r_* [ns^−1^]	*k_r_* [s^−1^]	*k_nr_* [ns^−1^]	*k_nr_* [s^−1^]
ACN	1.88	0.02	0.01	1.26 × 10^7^	0.52	5.19 × 10^8^
Butan-1-ol	1.68	0.11	0.07	6.56 × 10^7^	0.53	5.3 × 10^8^
Propan-1-ol	1.82	0.14	0.07	7.47 × 10^7^	0.47	4.75 × 10^8^
EtOH	1.70	0.19	0.11	1.12 × 10^8^	0.48	4.76 × 10^8^
MeOH	1.55	0.24	0.15	1.55 × 10^8^	0.49	4.9 × 10^8^
DMF	1.97	0.34	0.17	1.72 × 10^8^	0.34	3.35 × 10^8^
Glycerol	1.63	0.55	0.34	3.40 × 10^8^	0.27	2.73 × 10^8^
DMSO	2.10	0.49	0.23	2.33 × 10^8^	0.24	2.44 × 10^8^
**FTD-EtB**						
**Solvent**	**<τ>**	***Φ*_F_**	***k_r_* [ns^−1^]**	***k_r_* [s^−1^]**	***k_nr_* [ns^−1^]**	***k_nr_* [s^−1^]**
Propan-1-ol	0.18	0.07	0.40	4.01 × 10^8^	5.15	5.15 × 10^9^
Butan-1-ol	0.21	0.08	0.37	3.71 × 10^8^	4.39	4.39 × 10^9^
EtOH	0.18	0.09	0.48	4.84 × 10^8^	5.07	5.07 × 10^9^
ACN	0.21	0.09	0.44	4.35 × 10^8^	4.33	4.33 × 10^9^
MeOH	0.21	0.1	0.45	4.54 × 10^8^	4.31	4.31 × 10^9^
THF	0.20	0.1	0.49	4.87 × 10^8^	4.51	4.51 × 10^9^
Glycerol	0.27	0.20	0.76	7.55 × 10^8^	2.95	2.95 × 10^9^
DMSO	0.60	0.26	0.43	4.33 × 10^8^	1.23	1.23 × 10^9^

**Table 3:** The slope from Stokes shift versus bulk solvent polarity S_1_, spectral shift versus bulk solvent polarity function S_2_, Stokes shift versus microscopic polarity function m, Onsager cavity radius (a_0_), ground state (μ_g_), excited state (μ_e_) and change in dipole moment (Δμ) for compounds PhTD-ClB and FTD-EtB.

Compound	a_0_ [A]	S_1_	S_2_	m	μ_e_^a^ [D]	μ_g_^b^ [D]	μ_e_/μ_g_^c^	Δμ ^d^ [D]	Δμ ^e^ [D]	Number of Points	r^2^
PhTD-ClB	4.415	2478	−1873	3057	4.041	0.561	7.198	3.479	2.812	10:10 12	0.74; 0.71 0.58
FTD-EtB	4.353	5185	−2065	2805	4.654	2.003	2.323	2.651	2.637	11:9 12	0.70; 0.88 0.70

D = Debye = 3.33564 × 10^−30^ C m = 10^−18^ esu cm. ^a^ Experimentally calculated from the plot of Stokes shift vs. F_1_(ε, n). ^b^ Experimentally calculated from the plot of Stokes shift vs. F_2_(ε, n). ^c^ The ratio of μ_e_ and μ_g_. ^d^ The dipole moment change calculated from the experimental values of μ_e_ and μ_g_. ^e^ The dipole moment change calculated from the microscopic solvent polarity parameter ($E_{T}^{N}$).

**Table 4:** Results of the host-pathogen tests using a five-point scale.

*Blumeria graminis* f.sp. *avenae*										
	1 µg∙mL^−1^	2 µg∙mL^−1^	3 µg∙mL^−1^	4 µg∙mL^−1^	5 µg∙mL^−1^	6 µg∙mL^−1^	7 µg∙mL^−1^	8 µg∙mL^−1^	9 µg∙mL^−1^	10 µg∙mL^−1^
PhTD-ClB	4	4	4	4	4	4	4	4	4	4
FTD-EtB	0	0	0	0	0	0	0	0	0	0
***Blumeria graminis* f.sp. *Tritici***										
	1 µg∙mL^−1^	2 µg∙mL^−1^	3 µg∙mL^−1^	4 µg∙mL^−1^	5 µg∙mL^−1^	6 µg∙mL^−1^	7 µg∙mL^−1^	8 µg∙mL^−1^	9 µg∙mL^−1^	10 µg∙mL^−1^
PhTD-ClB	4	4	4	4	4	4	4	4	4	4
FTD-EtB	0	0	0	0	0	0	0	0	0	0
***Puccinia coronata* f.sp. *avenae***										
	1 µg∙mL^−1^	2 µg∙mL^−1^	3 µg∙mL^−1^	4 µg∙mL^−1^	5 µg∙mL^−1^	6 µg∙mL^−1^	7 µg∙mL^−1^	8 µg∙mL^−1^	9 µg∙mL^−1^	10 µg∙mL^−1^
PhTD-ClB	4	4	4	4	4	4	4	4	4	4
FTD-EtB	0	0	0	0	0	0	0	0	0	0
***Puccinia recondita* f.sp. *tritici***										
	1 µg∙mL^−1^	2 µg∙mL^−1^	3 µg∙mL^−1^	4 µg∙mL^−1^	5 µg∙mL^−1^	6 µg∙mL^−1^	7 µg∙mL^−1^	8 µg∙mL^−1^	9 µg∙mL^−1^	10 µg∙mL^−1^
PhTD-ClB	4	4	4	4	4	4	4	4	4	4
FTD-EtB	0	0	0	0	0	0	0	0	0	0

**Table 5:** A five-point scale for assessing oat leaf infection by fungal pathogens.

The Degree of Infection	Description of the Degree	Percentage Disease Control
**0**	Lack of symptoms	100%
**1**	The limited development of the pathogen visible single and small colonies	90%
**2**	Mycelium visible with a small quantity of spores—less than 20% of the leaf area	80%
**3**	Extensive mycelium occupying 20–50% leaf area	50%
**4**	Abundant mycelium occupying more than 50% of the leaf surface	0%

## Data Availability

The raw data supporting the conclusions of this article will be made available by the authors on request.

## Supplementary Materials

The following supporting information can be downloaded at https://www.mdpi.com/article/10.3390/ijms27177631/s1.
