# Supplementary material for: Aggregation-Modulated Excited-State Intramolecular Proton Transfer and Antifungal Potential of Selected 1,3,4-Thiadiazole Derivatives
Source: Int J Mol Sci. 2026 Aug 26;27(17):7631. doi: 10.3390/ijms27177631 (PMC13566912; doi:10.3390/ijms27177631)
Supplement: Supplementary file 1 [file ijms-27-07631-s001.zip › ijms-4487838-supplementary.pdf]

Supplementary materials

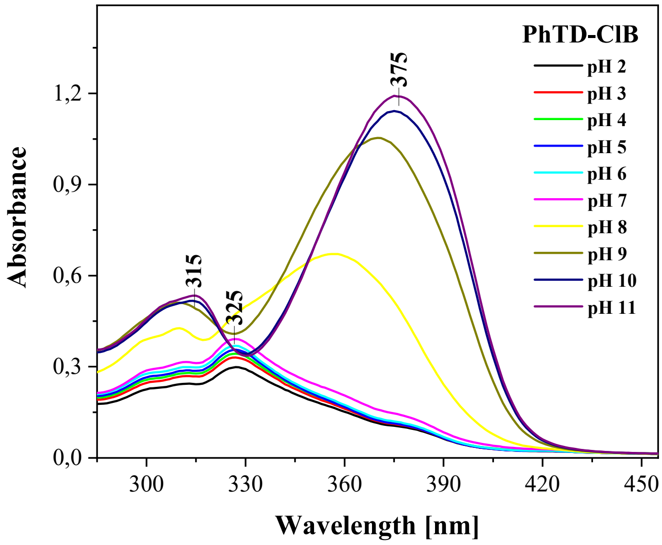

Figure S1. Electronic absorption spectra of PhTD-ClB at different pH values.

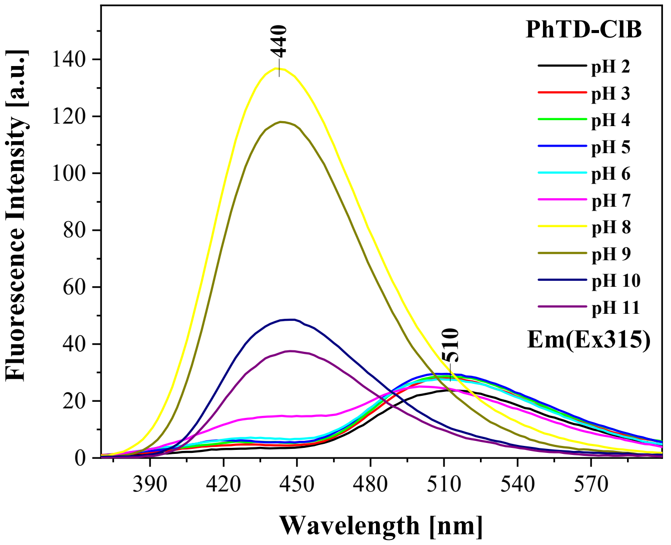

Figure S2. Fluorescence emission spectra of PhTD-ClB at different pH values at a wavelength of 315 nm.

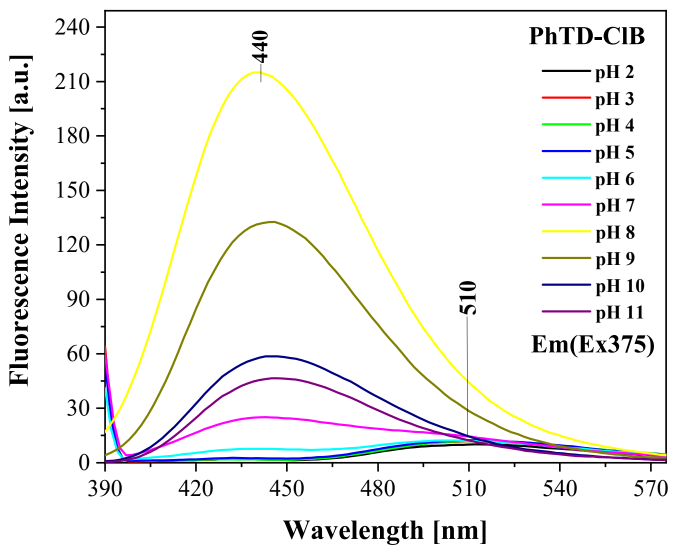

Figure S3. Fluorescence emission spectra of PhTD-ClB at different pH values at a wavelength of 375 nm.

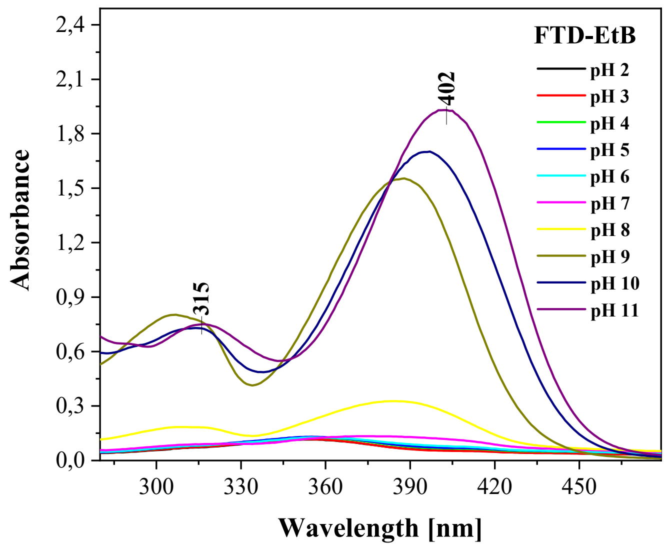

Figure S4. Electronic absorption spectra of FTD-EtB at different pH values.

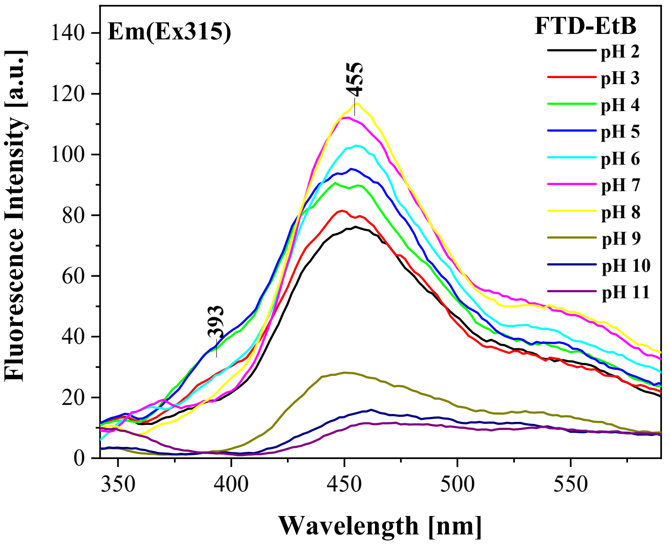

Figure S5. Fluorescence emission spectra of FTD-EtB at different pH values at a wavelength of 315 nm

A

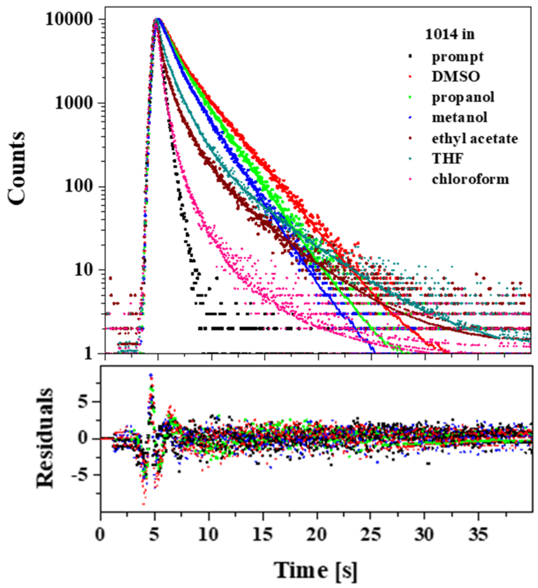

B

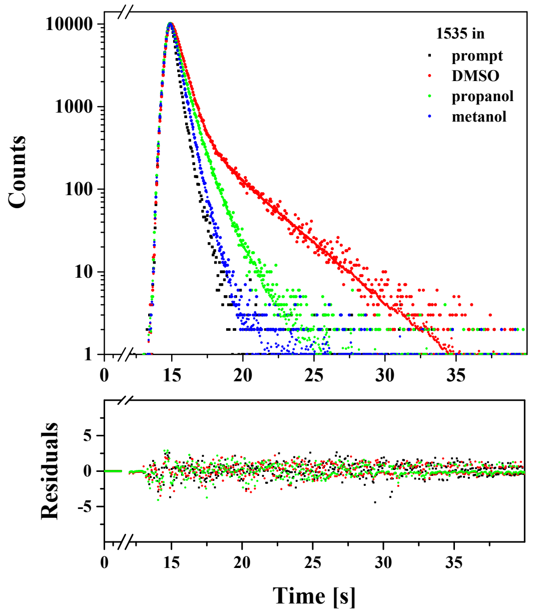

Figure S6. PhTD-ClB in solvents, decays with fit curves and residuals (panel A). FTD-EtB in solvents, decays with fit curves and residuals (panel B). The bottom panel shows residual distributions for the presented fits.

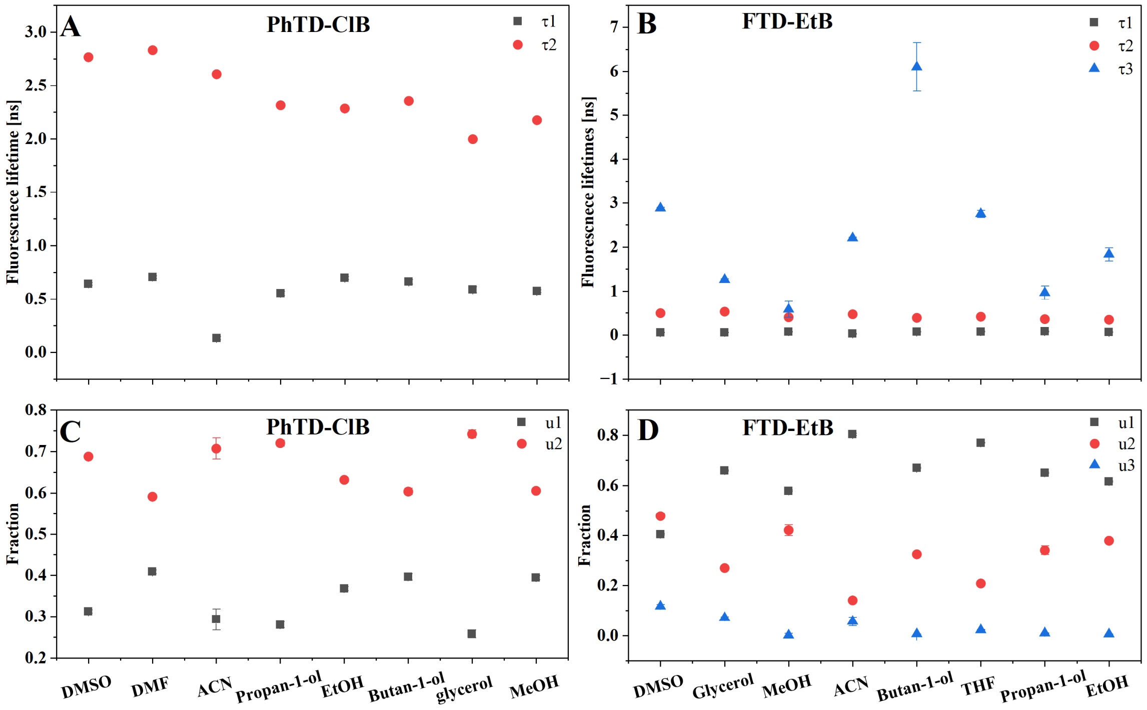

Figure S7. Measurements of PhTD-ClB and FTD-EtB fluorescence lifetimes (Panels A and B) and fractional intensities (Panels C and D) in solvents of varying polarity DMSO, DMF, ACN, propanol, ethanol, butan-1-ol, glycerol and methanol. Standard deviations indicated by bars.

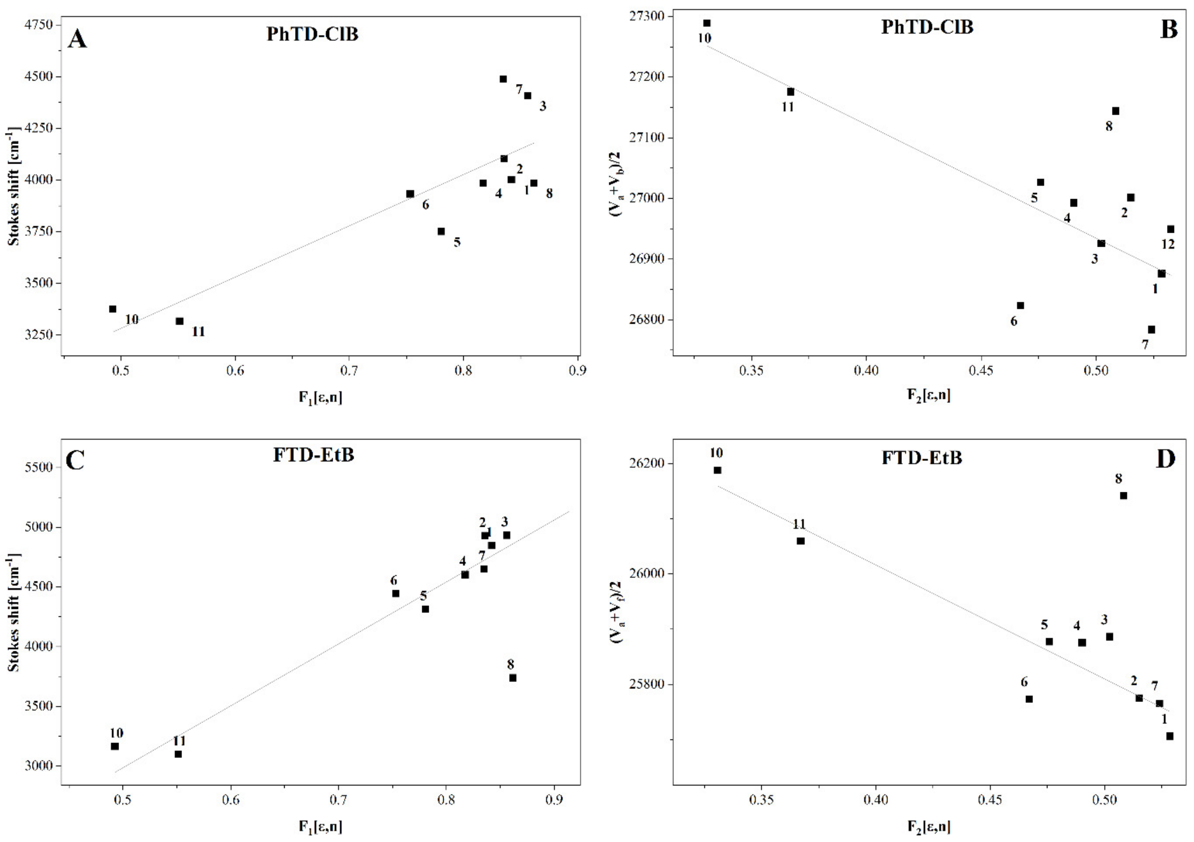

Figure S8. Stokes shift versus $F_1(\varepsilon, n)$ using Bakhshiev's equation (Panel A and C), $\frac{V_a+V_f}{2}$ versus $F_2(\varepsilon, n)$ using Kawski-Chamma-Viallet's equation (Panel B and D) for PhTD-ClB and FTD-EtB dissolved in different solvents (1 – DMSO, 2 – DMF, 3 – methanol, 4 – ethanol, 5 – propan-1-ol, 6 – butan-1-ol, 7 – glycerol, 8 – ACN, 9 – chloroform, 10 – ethyl acetate, 11 – THF and 12 – water).

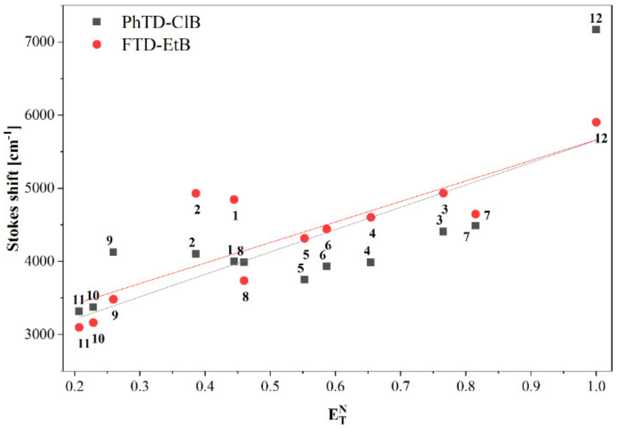

Figure S9. Stokes shift versus E_T^N for PhTD-ClB and FTD-EtB dissolved in different solvents (1 – DMSO, 2 – DMF, 3 – methanol, 4 – ethanol, 5 – propan-1-ol, 6 – butan-1-ol, 7 – glycerol, 8 – ACN, 9 – chloroform, 10 – ethyl acetate, 11 – THF and 12 – water).

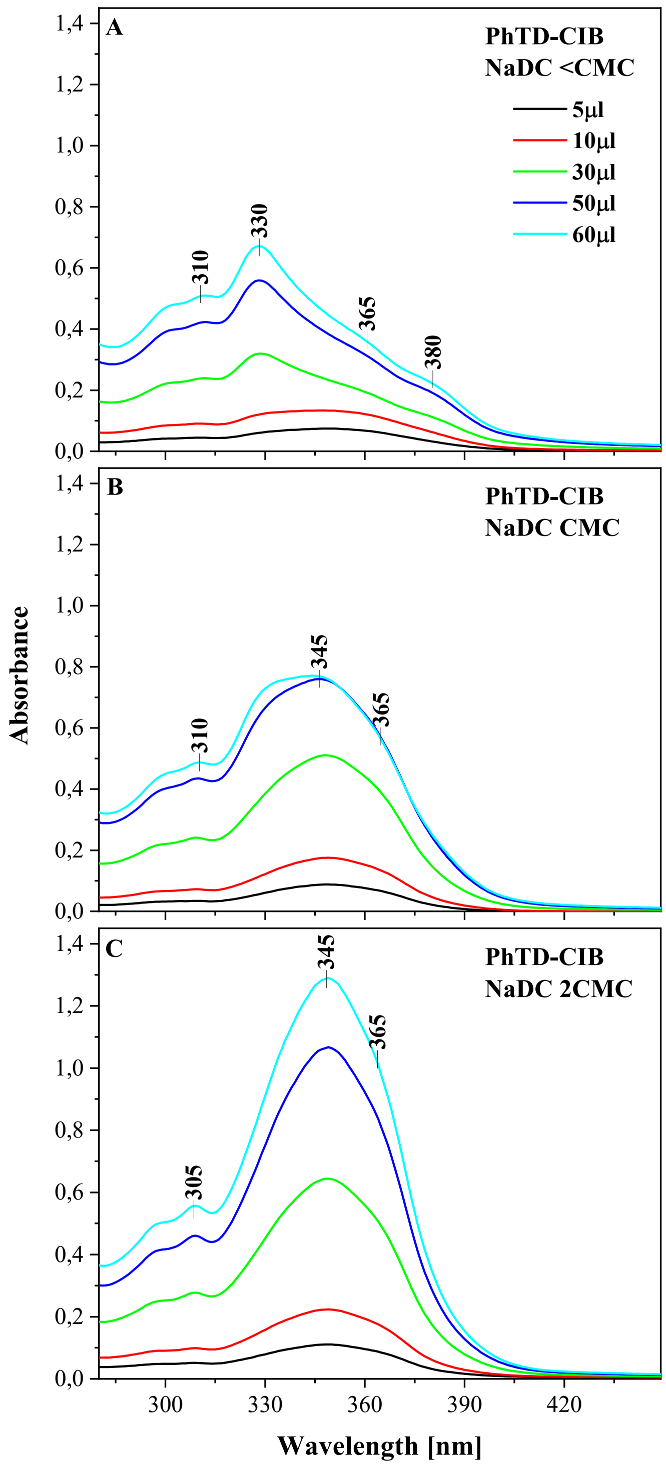

Figure S10. Electronic absorption spectra of PhTD-ClB in the micellar system at concentration <CMC (panel A), CMC (panel B) and 2CMC (panel C). The compound concentrations are respectively: 4.97×10^{-6}M, 9.9×10^{-6}M, 2.95×10^{-5}M, 4.89×10^{-5}M, 5.84×10^{-5}M.

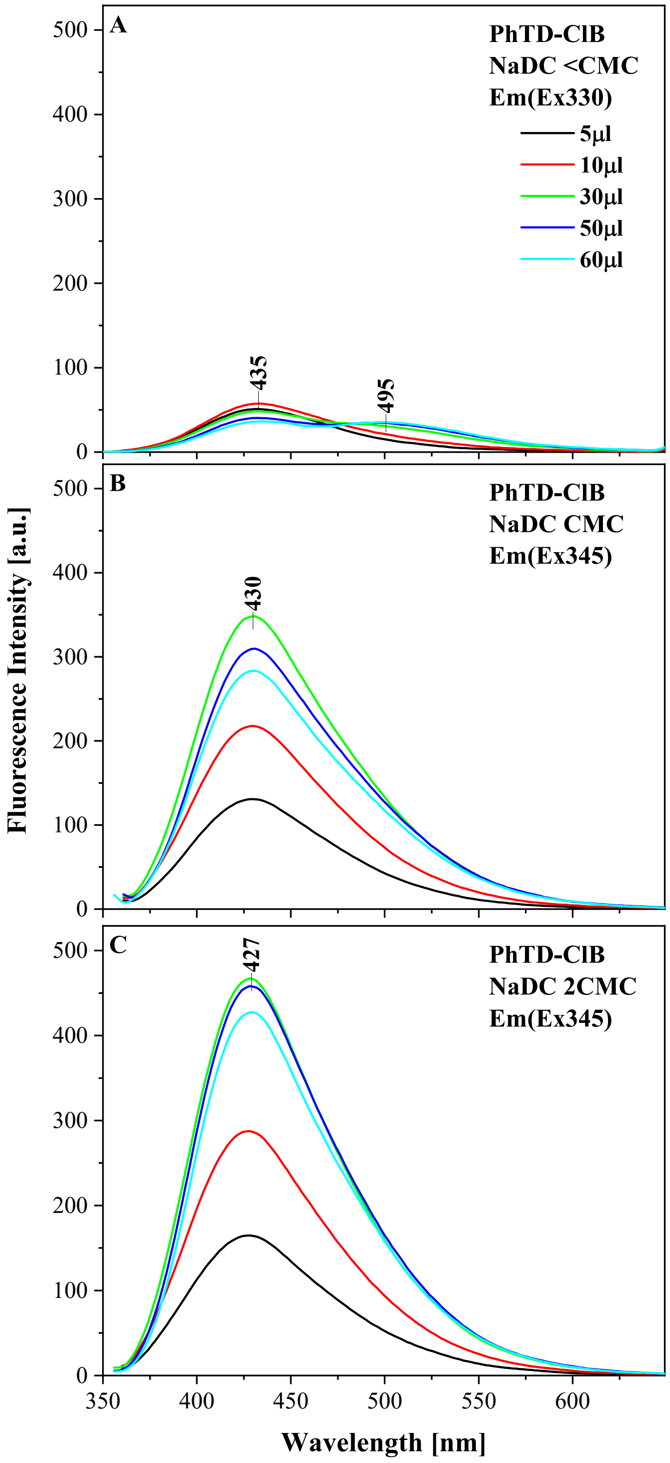

Figure S11. Fluorescence emission spectra of PhTD-ClB at excitation wavelengths of 345 and 330 nm in the micellar system with a concentration of <CMC (panel A), CMC (panel B) and 2CMC (panel C). The compound concentrations are, respectively 4.97×10^{-6}M, 9.9×10^{-6}M, 2.95×10^{-5}M, 4.89×10^{-5}M, 5.84×10^{-5}M.

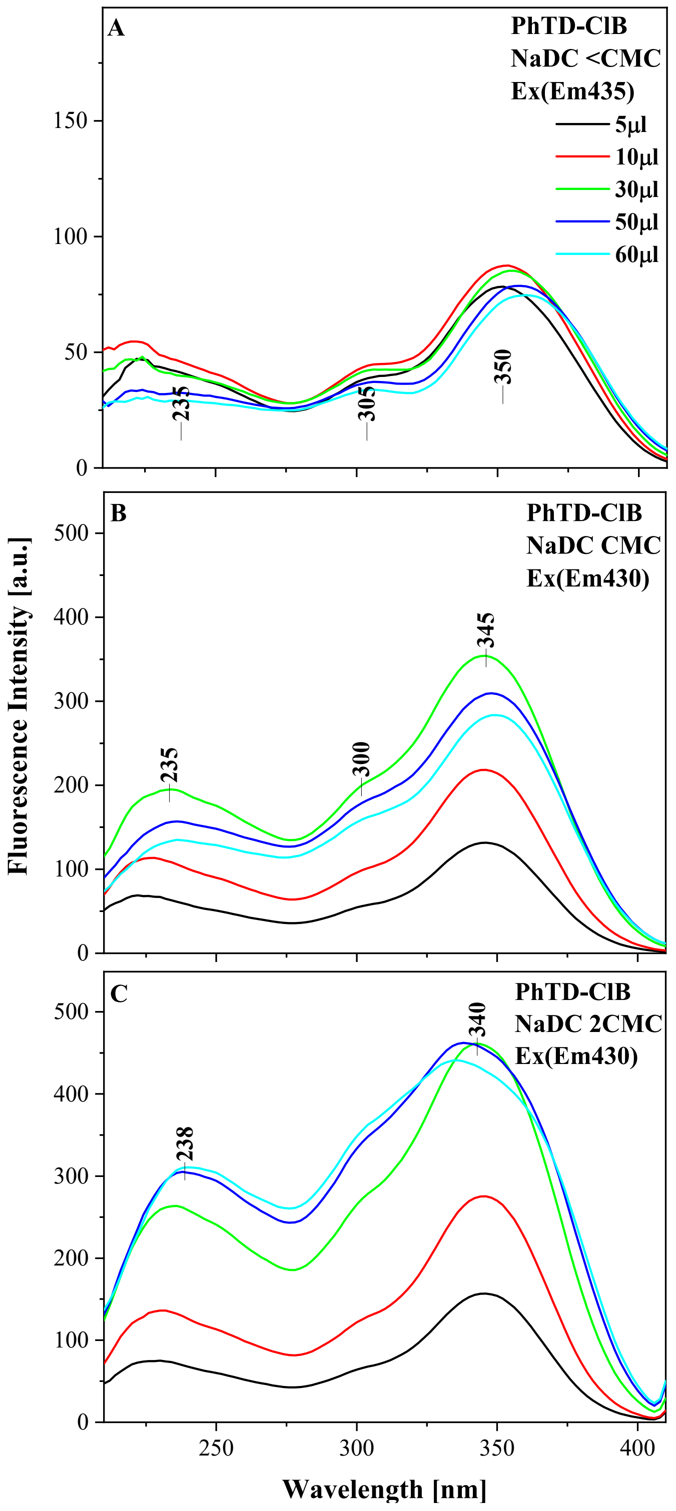

Figure S12. Fluorescence excitation spectra of PhTD-ClB at emission wavelengths of 430 and 435 nm in the micellar system with concentrations <CMC (panel A), CMC (panel B) and 2CMC (panel C). The compound concentrations are, respectively 4.97×10^{-6}M, 9.9×10^{-6}M, 2.95×10^{-5}M, 4.89×10^{-5}M, 5.84×10^{-5}M.

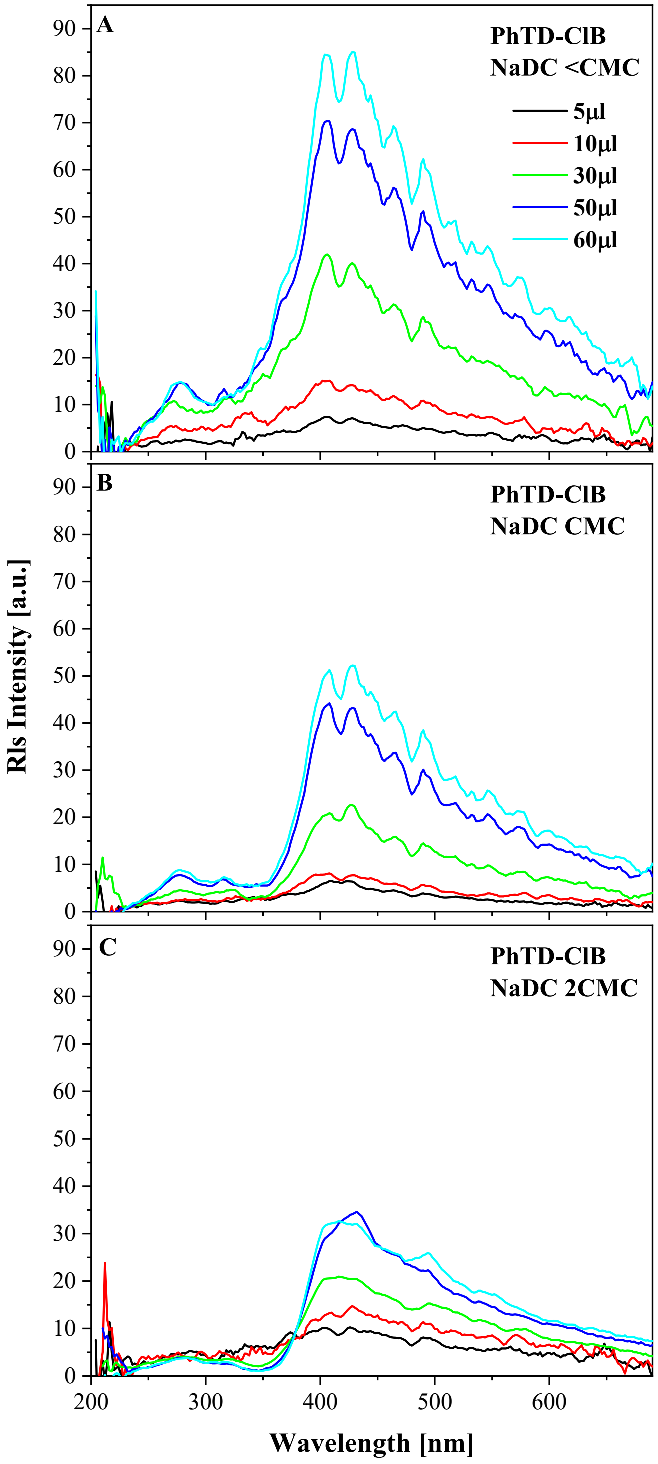

Figure S13. Resonance light scattering spectra of PhTD-ClB in the micellar system with concentration <CMC (panel A), CMC (panel B) and 2CMC (panel C). The compound concentrations are respectively 4.97×10^{-6}M, 9.9×10^{-6}M, 2.95×10^{-5}M, 4.89×10^{-5}M, 5.84×10^{-5}M.

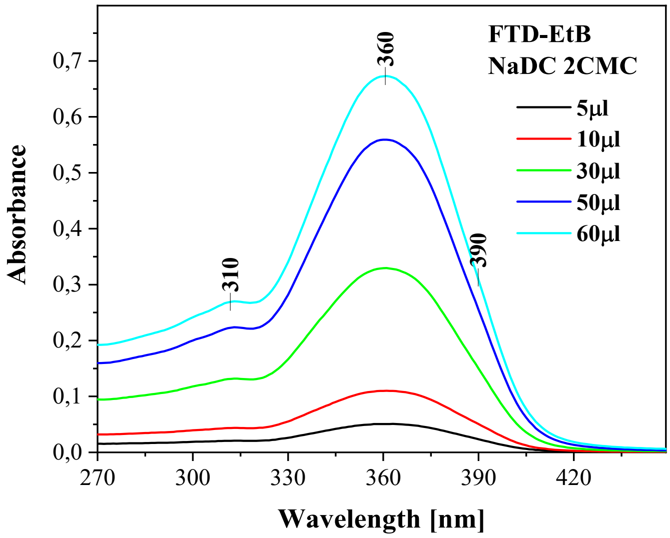

Figure S14. Electronic absorption spectra of FTD-EtB in a micellar system with a concentration of 2CMC. The concentrations of the compound are respectively 4.05×10^{-6}M, 8.06×10^{-6}M, 2.4×10^{-5}M, 3.98×10^{-5}M, 4.75×10^{-5}M.

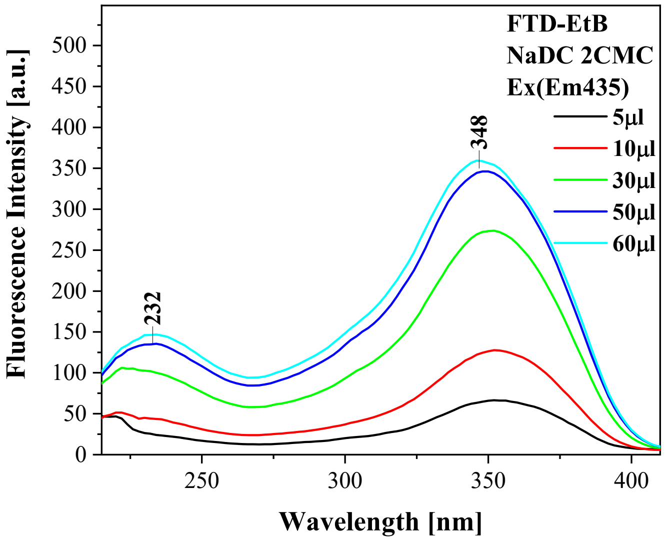

Figure S15. Fluorescence excitation spectra of FTD-EtB at an emission wavelength of 435 nm in a micellar system with a concentration of 2CMC. The compound concentrations are respectively 4.05×10^{-6}M, 8.06×10^{-6}M, 2.4×10^{-5}M, 3.98×10^{-5}M, 4.75×10^{-5}M.

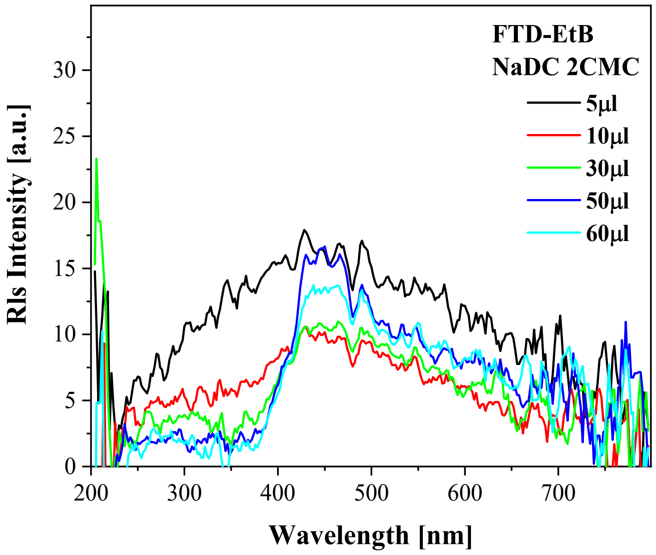

Figure S16. Resonance light scattering spectra of FTD-EtB in a micellar system with a concentration of 2CMC. The compound concentrations are respectively 4.05×10^{-6}M, 8.06×10^{-6}M, 2.4×10^{-5}M, 3.98×10^{-5}M, 4.75×10^{-5}M.

Table S1. Positions of the band maxima for PhTD-ClB and FTD-EtB compounds in selected solvents.

PhTD-ClB				
**Solvent**	**max Absorbance [nm]**	**max Absorbance [cm^{-1}]**	**max Fluorescence I [nm]**	**max Fluorescence II [nm]**
**MeOH**	343.3	29129.04	404.5	-
**EtOH**	345	28985.51	400	-
**Propan-1-ol**	346	28901.73	397.6	-
**Butan-1-ol**	347.35	28789.41	402.3	-
**Glycerol**	344.5	29027.58	407.5	-
**Water**	327.5	30534.35	428	508
**ACN**	343.2	29137.53	397.6	-
**Ethyl acetate**	345.1	28977.11	390.6	522
**THF**	346.8	28835.06	391.9	-
**Chloroform**	347	28818.44	405	509
**DMF**	344.2	29052.88	400.8	-
**DMSO**	346.3	28876.70	402	434
**FTD-EtB**				

Solvent	max Absorbance [nm]	max Absorbance [cm^{-1}]	max Fluorescence I [nm]	max Fluorescence II [nm]
**MeOH**	352.7	28352.71	427	-
**EtOH**	354.9	28176.95	424.2	-
**Propan-1-ol**	356.7	28034.76	421.6	-
**Butan-1-ol**	357.2	27995.52	424.6	-
**Glycerol**	356	28089.89	426.6	-
**Water**	354.9	28176.95	449	-
**ACN**	357	28011.20	412	527
**Ethyl acetate**	360.1	27770.06	406.4	549
**THF**	362.2	27609.06	408	-
**Chloroform**	362.6	27578.60	415	535
**DMF**	354.1	28240.61	429	-
**DMSO**	355.5	28129.40	429.5	-

Table S2. Dielectric constant ε, index of refraction n and solvent parameters $F_1(\varepsilon, n)$, $F_2(\varepsilon, n)$, $E_T(30)$, E_T^N for different solvents.

Solvent	ε	n	F_1 (ε, n)	F_2 (ε, n)	$E_T(30)$	E_T^N
DMSO	47.24	1.4773	0.842	0.529	55.5	0.765
DMF	36.7	1.4305	0.836	0.515	49.7	0.586
MeOH	33	1.3265	0.856	0.502	51.9	0.654
EtOH	25.3	1.3594	0.817	0.490	48.6	0.552
Propan-1-ol	20.18	1.3772	0.781	0.476	45.6	0.460
Butan-1-ol	17.8	1.3993	0.753	0.467	38.1	0.228
Glycerol	42.5	1.473	0.835	0.524	45.1	0.444
ACN	36.64	1.3416	0.862	0.508	43.2	0.386
Chloroform	4.81	1.4429	0.372	0.286	37.4	0.207
Ethyl acetate	6.08	1.3723	0.493	0.331	42.2	0.355
THF	7.6	1.404	0.551	0.367	33.9	0.099
Water	80.1	1.3333	0.914	0.532	39.1	0.259
